# Capture of fixation by rotational flow; a deterministic hypothesis regarding scaling and stochasticity in fixational eye movements

**DOI:** 10.3389/fnsys.2014.00029

**Published:** 2014-02-26

**Authors:** Nicholas M. Wilkinson, Giorgio Metta

**Affiliations:** ^1^iCub Facility, Fondazione Istituto Italiano di TecnologiaGenova, Italy; ^2^Centre for Robotics and Neural Systems, School of Computing and Mathematics, University of PlymouthPlymouth, UK

**Keywords:** fixational eye movement, tremor, traveling waves, spiral wave, phase singularity, Lévy walk, persistent neural activity, neural integrator

## Abstract

Visual scan paths exhibit complex, stochastic dynamics. Even during visual fixation, the eye is in constant motion. Fixational drift and tremor are thought to reflect fluctuations in the *persistent neural activity* of neural integrators in the oculomotor brainstem, which integrate sequences of transient saccadic velocity signals into a short term memory of eye position. Despite intensive research and much progress, the precise mechanisms by which oculomotor posture is maintained remain elusive. Drift exhibits a stochastic statistical profile which has been modeled using random walk formalisms. Tremor is widely dismissed as noise. Here we focus on the dynamical profile of fixational tremor, and argue that tremor may be a signal which usefully reflects the workings of oculomotor postural control. We identify signatures reminiscent of a certain flavor of transient neurodynamics; toric traveling waves which rotate around a central phase singularity. Spiral waves play an organizational role in dynamical systems at many scales throughout nature, though their potential functional role in brain activity remains a matter of educated speculation. Spiral waves have a repertoire of functionally interesting dynamical properties, including persistence, which suggest that they could in theory contribute to persistent neural activity in the oculomotor postural control system. Whilst speculative, the *singularity hypothesis* of oculomotor postural control implies testable predictions, and could provide the beginnings of an integrated dynamical framework for eye movements across scales.

## 1. Introduction

During fixation the eye is not still. Three main classes of *fixational eye movement* (henceforth FEM) have been identified (Martinez-Conde et al., 2004). Microsaccades are very fast movements which occur relatively infrequently. Drift is a slow, meandering component which occupies most of fixation time. Tremor is a fast, low amplitude aperiodic oscillation imposed on drift. Microsaccades are in many ways much like saccades on a tiny scale (Ko et al., 2010; Kagan and Hafed, 2013; Martinez-Conde et al., 2013; Otero-Millan et al., 2013; Poletti et al., 2013), though they may also be linked to the drift component (Engbert and Mergenthaler, 2006; Engbert et al., 2011). FEM have classically been thought to counteract sensory adaptation. Recent evidence suggests that FEM play a more sophisticated role, optimizing visual flow for the response properties of retinal ganglion cells (Rucci et al., 2007; Kuang et al., 2012) and relocating the highest resolution parts of the retina with great precision (Ko et al., 2010; Poletti et al., 2013). Some theories suggest that FEM perform an active perceptual palpitation of the visual scene which is fundamental to vision (Ahissar and Arieli, 2001, 2012; O'Regan and Noë, 2001). Recently, very high resolution eye movement data based on tracking tiny movements of ocular vein structure in three dimensions has revealed more structure to FEM than had previously been suspected (Li and Zhang, 2012; Zhang and Li, 2012). These studies reported microsaccades which were not straight and ballistic (as previously thought), but curving, and even bent and jerky. Relatively little detailed information was given, but it was reported that the drift-tremor combination took a complex, curling trajectory. These high resolution data may enable new insight into the underlying generative mechanisms of fixational eye movements. Oculomotor postural control is mediated by brainstem circuits (Aksay et al., 2000, 2007; Sparks, 2002) and is strongly associated with *persistent neural activity* (Major and Tank, 2004), which plays the role of *integrating* transient stimulation from superior colliculus reflecting saccadic velocity commands into persistent activity encoding the new eye position. The neuroanatomy and functional circuitry of oculomotor postural control has been intensively studied (e.g., Aksay et al., 2000, 2001, 2003; Miri et al., 2011a,b; Fisher et al., 2013), but the precise mechanisms underlying drift and tremor remain elusive.

Rotational waveforms (aka spiral waves, vortices, tori) are a commonplace, universal dynamical form which play an organizing role in dynamical systems at all scales, from galaxies to weather to evolution to organisms to organs to cells to photons (Toomre, 1969; Da-sheng, 1980; Boerlijst and Hogeweg, 1991; Gray and Jalife, 1996; Winfree, 2001; Molina-Terriza et al., 2007; Schecter et al., 2008; Taniguchi et al., 2013). This generality led Winfree (2001) to suggest toroidal temporal structure as a fundamental aspect of biological time, a notion for which the reference provides many empirical examples. Spiral waves are a canonical mode of pattern formation in dissipative systems operating far from equilibrium (Kuramoto and Koga, 1981; Cross and Hohenberg, 1993). The brain, by necessity, is one such system (e.g., Kelso, 1995; Ermentrout, 1998). Thus if toroidal waveforms (termed *spiral waves* in two dimensions, and *scroll waves* in three dimensions) do not play an organizing role in normal neurodynamics, then the brain must be considered something of an exception to the rule, which would require explanation. The suspected role of spiral waves in some pathological scenarios such as epilepsy (Milton, 2012) and cardiac fibrillation (Gray et al., 1998) suggests that the nervous system possesses mechanisms for actively suppressing turbulence and spatiotemporal chaos (e.g., Schiff et al., 1994), but observations of spiral waves in non-pathological settings (Jung et al., 1998; Huang et al., 2004, 2010) clarify that such suppression is not complete or universal. Indeed, an active field of study in cardiac defibrillation is the suppression of spatiotemporal chaos and turbulent neural activity by the seeding of spiral waves (e.g., Zhang et al., 2002; Xiao-Ping et al., 2011).

The current contribution hypothesizes a connection between quasi-persistent spiral neurodynamics and *persistent neural activity* in the context of oculomotor postural control (Major and Tank, 2004). Sections 2 and 3 respectively introduce the literature on spiral waves and fixational eye movements. Section 4 details our motivations in proposing the *singularity hypothesis* of postural memory. Our purpose is not an exhaustive review, nor to convince the reader that our hypothesis is necessarily correct, but a targeted presentation of empirical evidence and functional arguments which render the singularity hypothesis interesting, plausible and worth testing. Section 5 offers some concluding remarks. Predictions are presented in boxes in the main text.

## 2. Transient neurodynamics and spiral waves

The classical focus on attractor networks in systems neuroscience (see for review Amit, 1992) is increasingly being enriched by a modern synthesis which also stresses the importance of self-organization and transient neural dynamics (Rabinovich et al., 2001; Maass et al., 2002; Seliger et al., 2003; Durstewitz and Deco, 2008; Friston et al., 2012; Milton, 2012), fractality in physiology (Goldberger and West, 1987; West et al., 1994; Werner, 2010; West, 2010), self-organizing criticality (Bak et al., 1987; Bak, 1996; Jung et al., 1998), chaotic itinerancy (Tsuda, 1991, 2001; Kaneko and Tsuda, 2003) and dynamic pattern formation in non-equilibrium dissipative systems (Cross and Hohenberg, 1993).

Neural spiral waves are an intriguing class of quasi-persistent transient neurodynamics, whose functional potential in brain activity remains an open question. They have received extensive theoretical attention in terms of their abstract properties in networks (e.g., Coombes, 2005; Kilpatrick and Bressloff, 2010b; Ma et al., 2012b), but surprisingly little attention in terms of concrete cases linking their dynamics to perception and behavior. We have conducted preliminary modeling studies employing spiral waves for visual salience mapping (Wilkinson and Metta, 2011; Wilkinson et al., 2011), and spiral neurodynamics have linked to visual geometric hallucination (Bressloff et al., 2001; Kilpatrick and Ermentrout, 2012a,b; Froese et al., 2013). At the motor end, Heitmann, Breakspear and colleagues have developed physiologically explanatory models showing how traveling waves (including spirals) can encode motor trajectories (Heitmann, 2013).

###  

#### 2.0.1. Spiral waves in nature, biology, and the brain

The multiscale ubiquity of spiral waves in nature and biology (Toomre, 1969; Lechleiter et al., 1991; Winfree, 2001), and their interesting dynamical properties (Boerlijst and Hogeweg, 1991; Biktashev and Holden, 1993, 1995; Langham and Barkley, 2013), have motivated many physical, chemical, and mathematical studies. Arthur Winfree pioneered computational and empirical investigations of toroidal dynamics in chemical and biological systems (Winfree, 1967, 1972). Many biological dynamics exhibit toroidal form (Winfree, 2001). The modern understanding of pathological heart fibrillation (and de-fibrillation intervention) is perhaps the most prominent medical application of this work (e.g., Gray et al., 1998; Gray and Chattipakorn, 2005), though cellular calcium dynamics is another important example (Lechleiter et al., 1991). Spirals are reentrant waves which circle around a central rotor known as a *phase singularity* (Winfree, 1991); a point of maximally uncertain phase, surrounded by points of all phases. The central rotor of a whirlpool or tornado provides a physical example in three dimensions.

Propagating calcium waves in astrocyte networks are thought to play an important role in regulating brain activity (Cornell-Bell and Finkbeiner, 1991; Finkbeiner, 1992). Jung et al. (1998) observed that *Ca*^2+^ spiral waves exhibiting scale-free distributions suggestive of self-organizing criticality (Bak et al., 1987) are characteristic of healthy function, whilst epileptic events are characterized by the breakdown of this scaling. In neural tissue, traveling waves have been observed widely in various species in both sensory and motor cortices (see for review Wu et al., 2008; Sato et al., 2012) via voltage sensitive dye imaging (“VSDI”). VSDI is an invasive optical imaging method which enables measurement of subthreshold changes in membrane potential with high spatiotemporal resolution (Grinvald and Hildesheim, 2004). Spiral dynamics are commonplace in the dynamics of simulated excitable media including networks of model neurons (Milton et al., 1993; Winfree, 2001; Chun-Ni et al., 2010; Yu et al., 2010; Ma et al., 2012a), and have been observed in mammalian (Huang et al., 2004, 2010) and reptilian (Prechtl et al., 1997) cortex. Movies of cortical spiral waves in the VSDI signal (from Huang et al., 2010) can be found http://www9.georgetown.edu/faculty/wuj/propagationwave.html.

It has been suggested that spiral waves may play an organizing role in neural field interactions (Wu et al., 2008; Freeman, 2009; Huang et al., 2010). Short-lived spiral waves are frequently observed in the healthy case (Huang et al., 2010), but the growth of spiral wave formations of large duration and extent has been linked to pathological conditions including heart fibrillation (Gray et al., 1998) and epileptic seizure (Milton and Jung, 2003; Viventi et al., 2011; Milton, 2012; Stacey, 2012). This is suggestive that spiral waves are a part of normal function, whether constitutive or epiphenomenal, but that their (potentially useful) tendency to enslave surrounding dynamics (e.g., Savill et al., 1997; Yang and Yang, 2007; Huang et al., 2010) has to be carefully controlled.

#### 2.0.2. Dynamical behavior of spiral waves

The dynamical behavior of spiral waves can be complex and is the subject of extensive research. A useful introduction with video visualizations is given at Björn Sandstede's website hosted by the Department of Applied Mathematics at Brown University, USA, http://www.dam.brown.edu/people/sandsted/research.php?project=spirals. At the risk of oversimplifying, the behavior of spiral singularities exhibits three basic components. Firstly, the rotational orbit of the whole spiral wave will exhibit a characteristic frequency and phase, and is reflected in a small, “on the spot” circular rotation of the phase singularity at the spiral tip. The current hypothesis proposes that this component may correspond to the low frequency peak observed in tremor statistics (Spauschus et al., 1999; Greschner et al., 2002), and could explain new high resolution observations of curling trajectories of drift and tremor (Li and Zhang, 2012; Zhang and Li, 2012).

**Figure 1:**
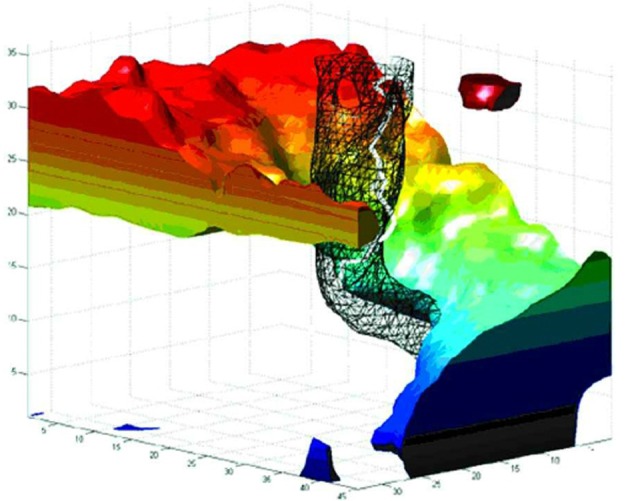
**The evolution of a spiral wave over one rotational orbit**. Space is represented in the horizontal axes, and time (in milliseconds) on the vertical axis. The black mesh encloses a thresholded area of reduced variance (i.e., low amplitude) at the spiral center, as observed in cortex by Huang et al. (2004, 2010). The white tube within the black mesh tracks the evolution of the phase singularity at the spiral core. Note the fast (80–90 Hz) oscillation of the singularity, which we hypothesize underlies the fast component of fixational tremor. Reproduced from Figure 8B in Bray et al. (2001), copyright John Wiley and Sons Publishing 2001.

**Figure 2:**
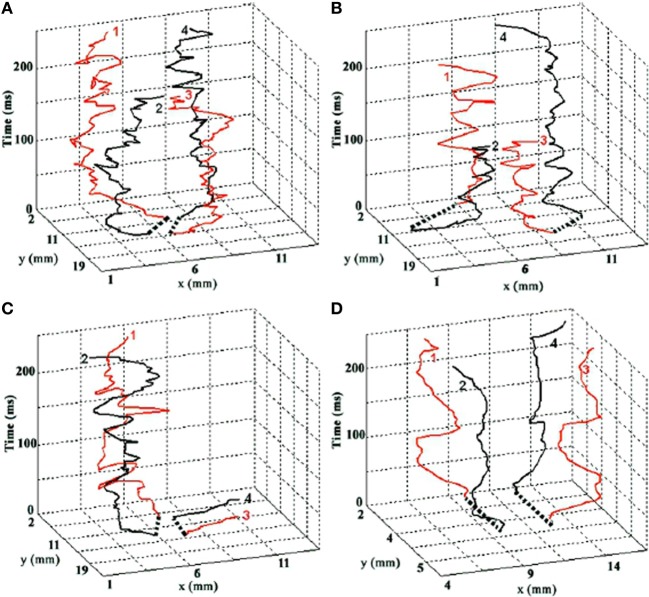
**The evolution of four interacting spiral singularities in space (horizontal axes) over time (vertical axis)**. Time is denoted in milliseconds. Note the fast (80–90 Hz) oscillation of the singularities, which we hypothesize underlies the fast component of fixational tremor. Graphs **A–C** are real trajectories from cardiac data. Graph **D** is from a computational model. Reprinted from Figure 4 in Bray et al. (2001), copyright John Wiley and Sons Publishing 2001.

**Figure 3:**
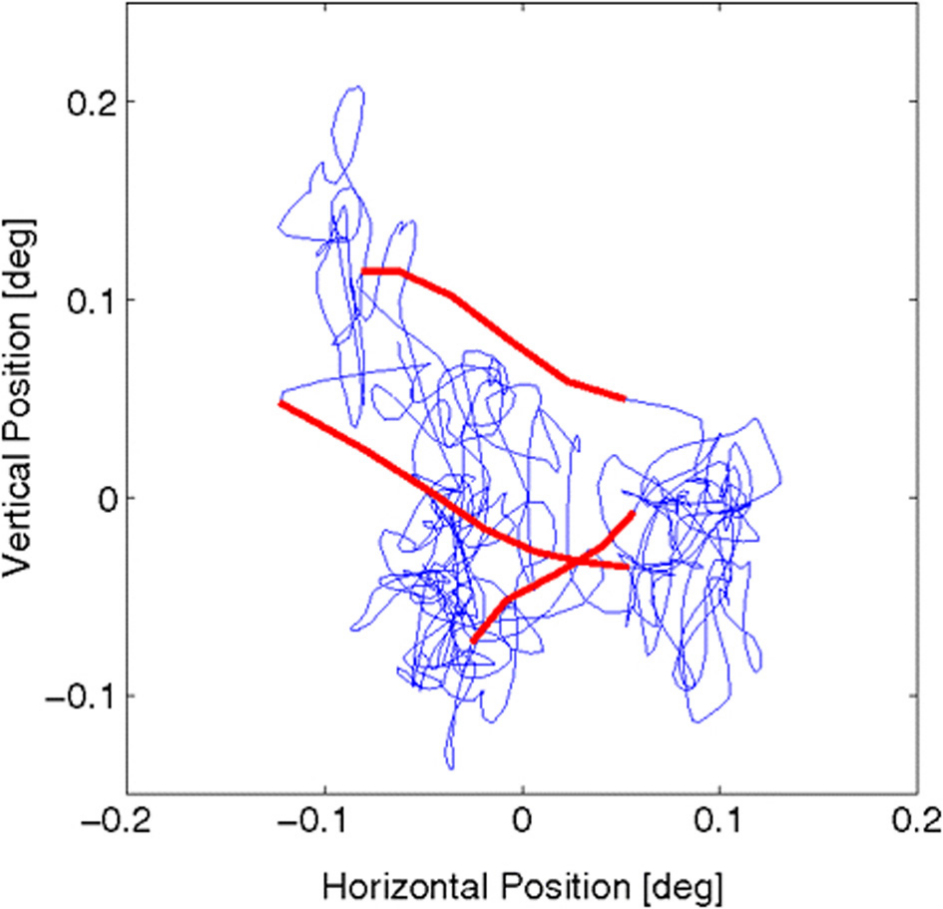
**Fixational eye movements and microsaccades, from Figure 1 in Engbert et al. (2011)**. Data were recorded from fixational eye movements during a fixation of 2 s. Slow movements (blue) are highly erratic, whereas microsaccades (red) are ballistic, small-amplitude epochs with a more linear trajectory (compared with the slow background motions). The sample trajectory was recorded with a sampling frequency of 500 Hz (for details see ref. 29 in Engbert et al., 2011). Reprinted from Engbert et al. (2011), copyright PNAS 2011.

**Figure 4:**
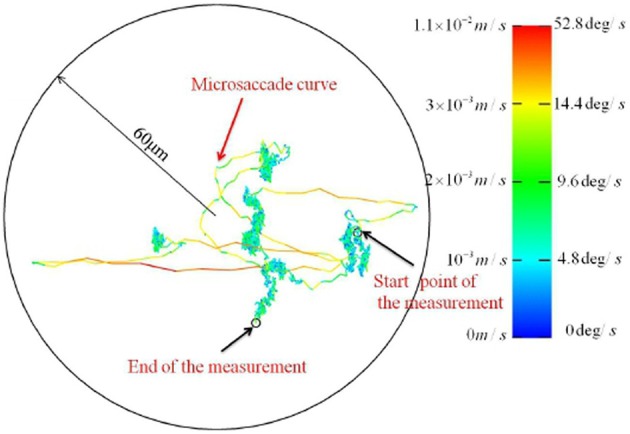
**FEM trajectories recorded with a new high resolution video tracking technique**. [Adapted from Zhang and Li, 2012].

**Figure 5:**
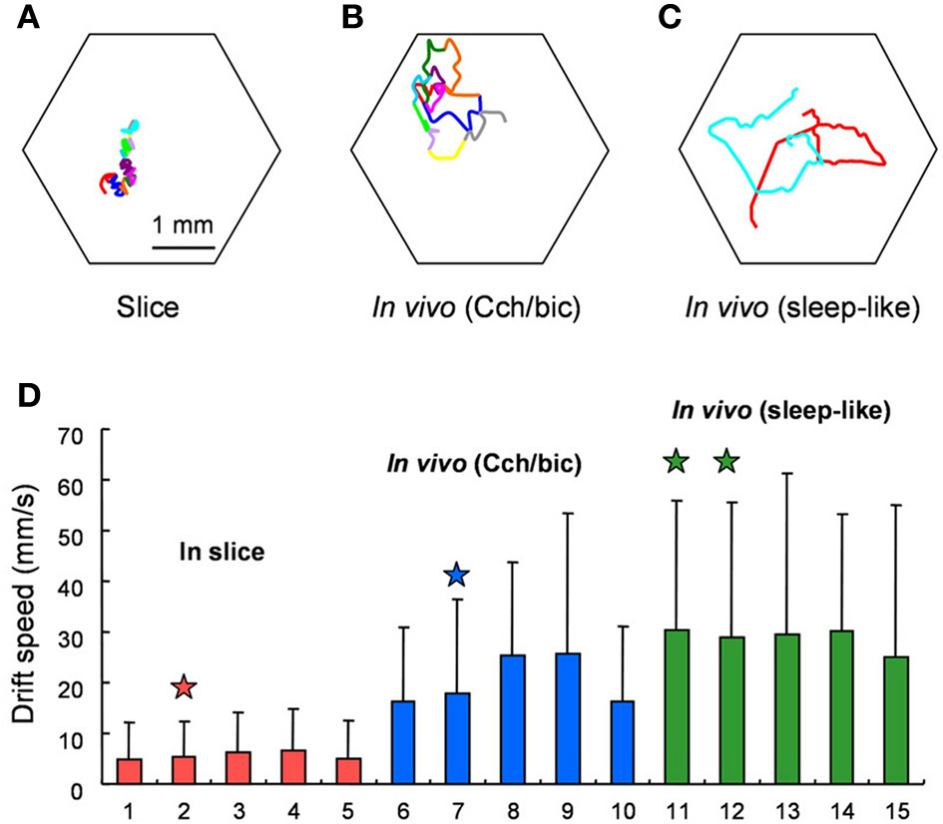
**Behavior of spiral waves in mammalian visual cortex under various conditions**. From the original; Drifting of Spiral Phase Singularities. **(A)** Trajectory of spiral phase singularity during a 12-cycle spiral waves in cortical slices. **(B)** Trajectory of spiral phase singularity during an 11-cycle spiral waves *in vivo* under Cch/bic application. Hexagon shows the field of view and each color represents one cycle of spiral wave. **(C)** Trajectory of spiral phase singularities during 2 spiral waves (red and cyan, each with 1.5 turn) during sleep-like states. **(D)** Comparison of drifting speed of spiral phase singularity for slices and *in vivo*. Five examples from *in vivo* under Cch/bic, *in vivo* during sleep-like states and slices, respectively, are shown (mean + *SD*). Columns with stars on top are from the examples in **(A,C)**. The standard deviation is large because the drifting of spiral phase singularity is not consistent and there are large variations from time to time. The difference between *in vivo* and slices is statistically significant (Welch's test, *p* < 0.001, 25 *t* tests). The difference between *in vivo* (Cch/bic) and *in vivo* (sleep-like) is also significant. Reprinted from Figure 7 in Huang et al. (2010), copyright Elsevier 2010.

**Figure 6:**
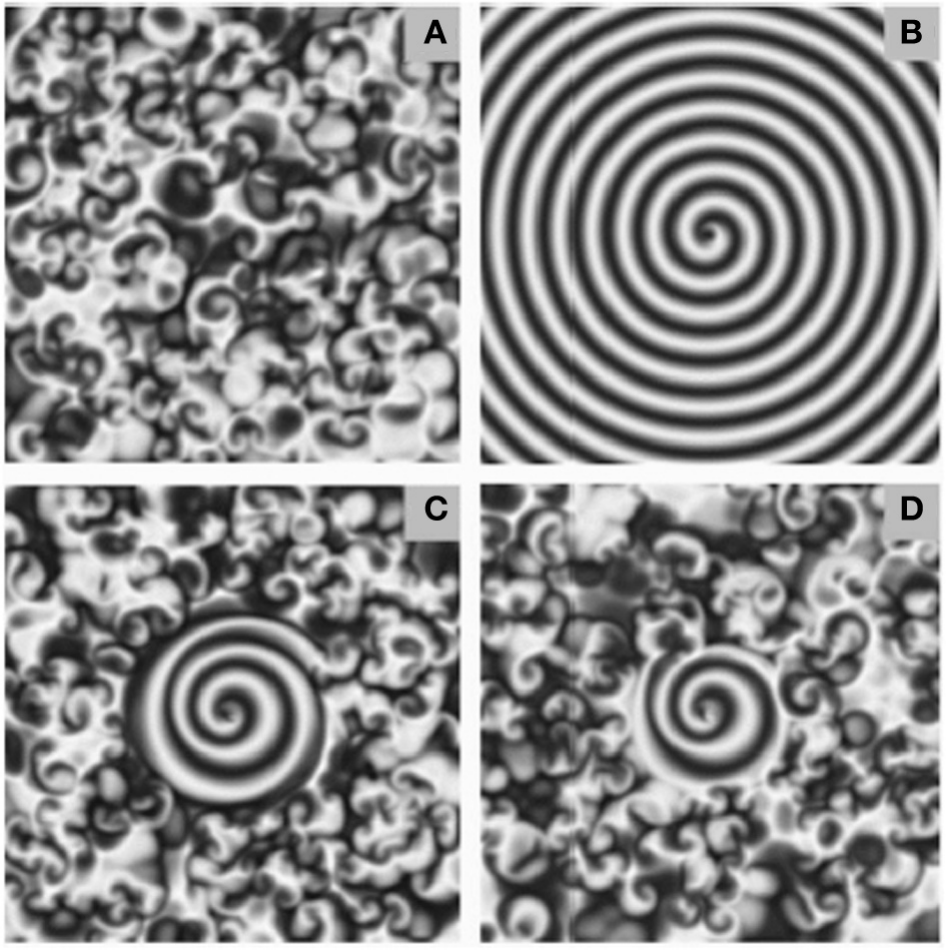
**Examples of spiral seeding in a background of chaotic turbulence**. Seeding and growing spiral waves in a background of chaotic turbulence. The extent of the spiral wave seeded was dependent on the frequency of periodic forcing at the singularity. Too low, or too high, frequency was less effective. The ratio of the frequency of the forcing signal to that of the angular frequency of the spiral equalled **(A)** 0.6 **(B)** 0.8 **(C)** 1.0 **(D)** 1.2. Figure reprinted from Zhang et al. (2002). Copyright American Physical Society 2002.

**Figure 7:**
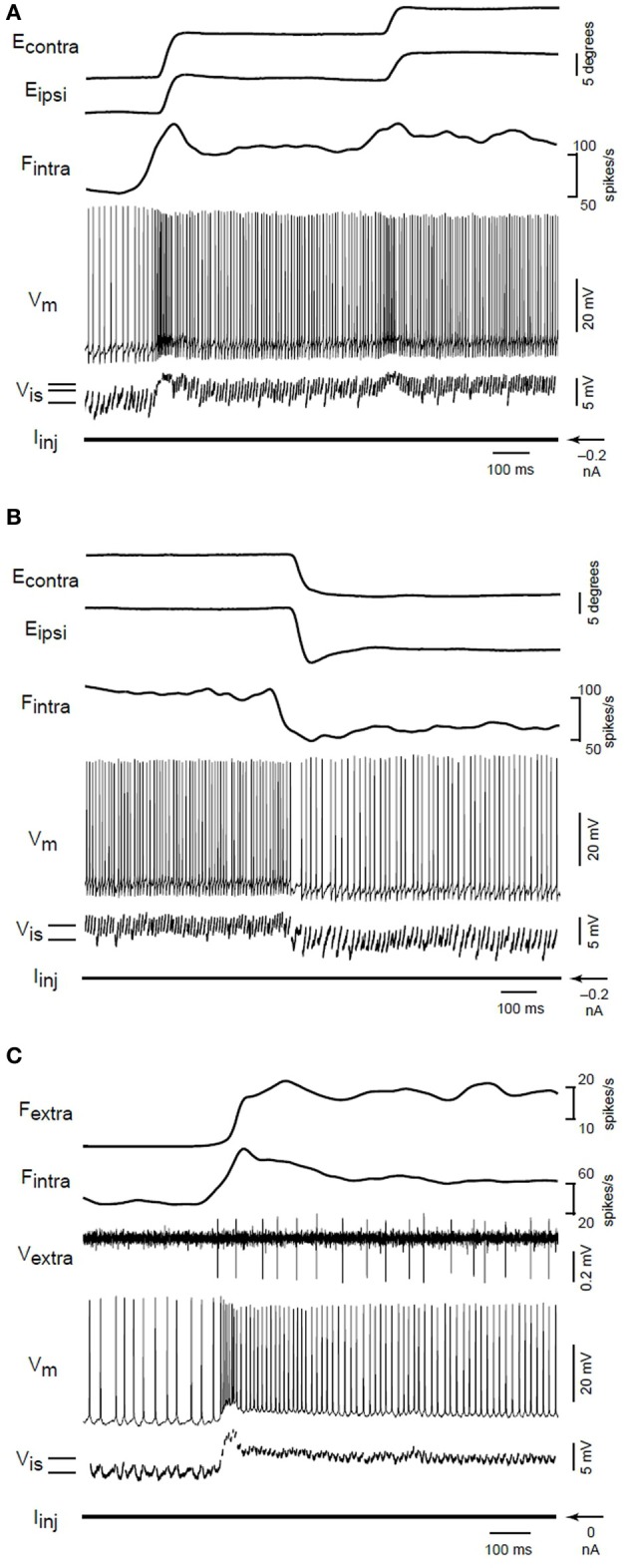
**From the original; Membrane potential changes during transitions in fixation position**. **(A)** Eye position, firing rate of an intracellularly recorded neuron (*F*_intra_), membrane potential (*V*_*m*_), interspike membrane potential (*V*_*is*_), and injected current (*I*_inj_) for on-direction steps during intracellular recording. Solid lines at lower left indicate the average value of *V*_*is*_ during separate fixations. **(B)** Changes during off-direction steps of the ipsilateral eye. This segment is taken from the end of a nasaltemporalnasal cycle that started with the transitions shown in **(A)**. **(C)** Rate and potential changes for a different neuron during an on-direction step. The firing rate (*F*_extra_) of a second extracellularly recorded position neuron (*V*_extra_) served as a surrogate for eye position. In this recording, the fast afterhyperpolarization following action potentials was abolished by substituting cesium for potassium in the electrode solution. From Aksay et al. (2001), copyright Nature Publishing 2001.

**Figure 8:**
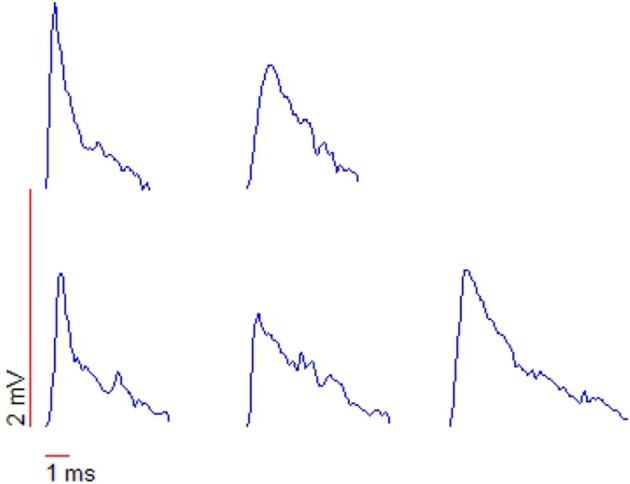
**Shapes of action potentials recorded with whole-cell patch measurements**. Note the “peculiar” triangular form, typical of the leading edge of a spiral waveform. From Huang (2009), copyright The Author.

**Figure 9:**
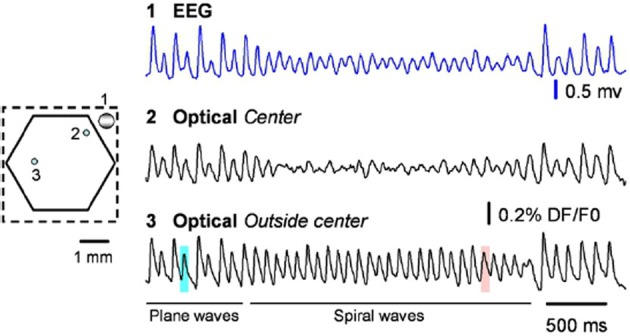
**From the original; raw data traces from the EEG (blue) and two optical detectors labeled on the left**. Detector 2 (Optical spiral center) is selected from the spiral center, showing large amplitude reduction. Detector 3 is selected from a location that spiral center never swept through, showing no amplitude reduction. From Huang et al. (2010), copyright Elsevier 2010.

**Figure 10:**
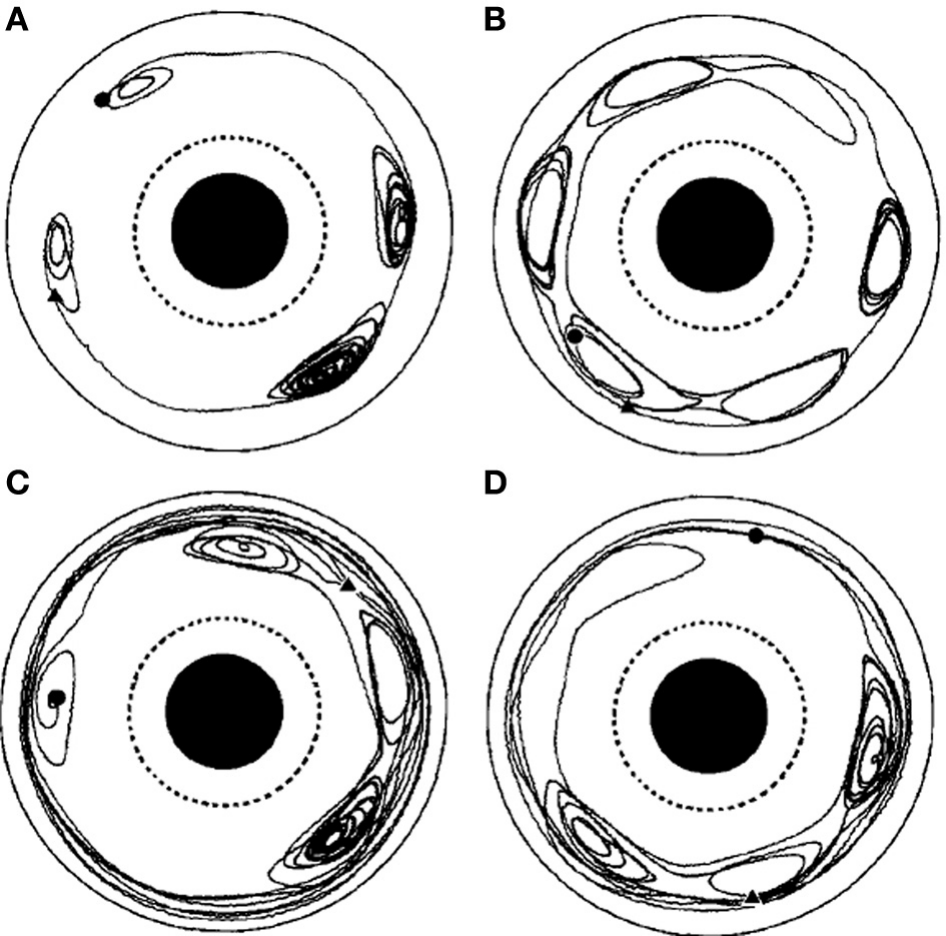
**From the original; Chaotic particle trajectories in a time-periodic flow**. **(A–D)** Depict different runs of the apparatus with different numbers and locations of vortices. From the original; Chaotic particle trajectories in a time-periodic flow. Long sticking events can be seen in each case, and flights of length greater than one rotation about the annulus can be seen in **(C)**, **(D)**. Hyperbolic fixed points, near which the particle motion is particularly sensitive to transitions between flights and sticking events, are evident in all the trajectories. The particle motion is viewed from a reference frame that is co-rotating with the vortex chain, and the beginning of each trajectory is marked by a circle, the end by a triangle. From Solomon et al. (1994), copyright American Physical Society 1994.

**Figure 11:**
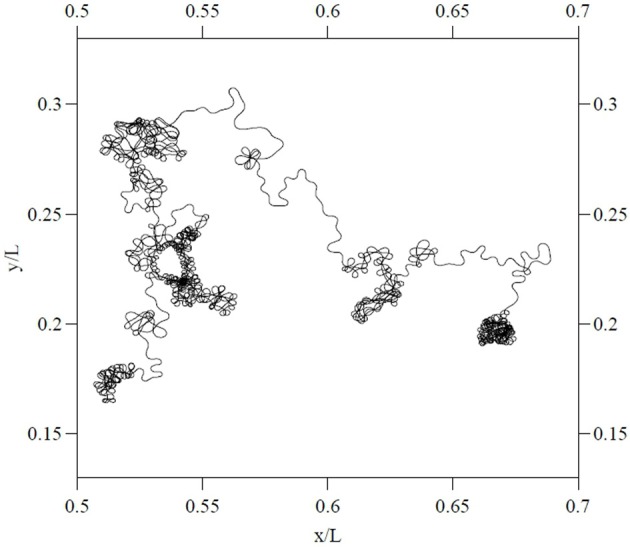
**Particle trajectory in a model of two dimensional magnetic turbulent flow**. From Chiaravalloti et al. (2006), copyright the Royal Swedish Academy of Sciences 2006. Reproduced by permission of IOP publishing.

This trajectory may be perturbed in various ways to take on a locally more complex, globally drifting form. This is known as spiral *drift*, and occurs in response to various forms of symmetry breaking perturbations/gradients in the external milieu (Biktashev and Holden, 1995; Wulff, 1996; Sandstede et al., 1999; Biktashev, 2007). Figures 1–11 in the Scholarpedia article Biktashev (2007) (http://www.scholarpedia.org/article/Drift_of_spiral_waves), display images and animated movies of the trajectory of spiral singularities under various forms of symmetry breaking. Note the basic curling trajectory, whose period is equal to that of the wave's orbit. This spiral drift in response to symmetry breaking perturbations in the excitability of the medium is the neural correlate we hypothesize for the well known slow component of fixational drift (Martinez-Conde et al., 2004; Rolfs, 2009).

In addition to the above relatively slow components, fast, aperiodic oscillatory modulations of the basic curling trajectory can result from instabilities at the phase singularity (Winfree, 1991). The singularity is the point at which all surrounding signals cancel exactly, and so small fluctuations in the surround cause this point of balance to jitter unpredictably. Gray et al. (1998); Bray et al. (2001) tracked the spacetime trajectory of phase singularities in cardiac fibrillation data. Figure 1, from Bray et al. (2001) depicts the evolution of a real cardiac phase singularity (white tube inside black mesh) in detail over one cycle of the carrying spiral wave. Figure 2, also from Bray et al. (2001) graphs longer trajectories of the singularities of four interacting spirals. Note the fast (80–90 Hz) aperiodic oscillation superimposed on the basic curling trajectory, much faster than the period of the carrier wave. Gray et al. (1998) reported similar spiral meander during cardiac fibrillation. Though here in cardiac tissue, this instability at the singularity is a universal feature of spiral waves (Winfree, 1991). The current proposal suggests this instability as the source of the well known high frequency component of fixational tremor (Martinez-Conde et al., 2004; Rolfs, 2009).

#### 2.0.3. The functional role of neural traveling waves

Traveling waves are routinely observed throughout the brain (Wu et al., 2008), and evidence is increasingly suggesting that they play a functional role (Modolo et al., 2011; Sato et al., 2012; Bahramisharif et al., 2013). Heitmann, Breakspear and colleagues have produced a series of physiologically explanatory and plausible models showing how traveling waves can encode motor trajectories read out by dendritic spatial filters (Breakspear et al., 2010; Heitmann et al., 2012, 2013). These are particularly interesting in the current context. In these models, traveling waves encode motor patterns defining *movement*, whilst synchrony constitutes the *resting state*. The current model, in which spiral waves encode for the active holding of posture, sits well in this framework, because spirals, unlike other traveling waves, *have and (almost) hold a location* in a specific sense (Biktasheva and Biktashev, 2003; Langham and Barkley, 2013). This makes them interesting for the kind of active almost-stillness characterizing postural control.

Spiral wave activity has been observed in the VSDI signal, which primarily reflects the field dynamics of sub-threshold membrane potentials (Grinvald and Hildesheim, 2004). These waves can keep cells in a depolarized “ready” state for input, or indeed polarize cells to effectively ignore input (Bahramisharif et al., 2013). This implements a form of spatiotemporally structured *gain control*, widely agreed to be a fundamental aspect of nervous function (Hillyard et al., 1998; Salinas and Thier, 2000; Salinas and Sejnowski, 2001; Rothman et al., 2009; Olsen et al., 2012). Gain fields have been associated with attentional selectivity at both the sensory and motor end (Aston-Jones and Cohen, 2005; Saalmann and Kastner, 2009; Sara and Bouret, 2012).

On this view, fast, aperiodic spiral meander depolarizes a point locus of local cells in the *gamma* band (peaking around 80–90 Hz) in a quasi-phaseless manner. The region outside the spiral center is polarized and depolarized periodically by the spin of the spiral arms, on a slower (5–25 Hz) scale dependent on the period of the spiral orbit and the number of spiral arms. Examining the relationship between local field potential and spike rates in the temporal cortex, Zanos et al. (2012) found two populations of cells with just these response characteristics. One population responded at high frequencies in a phase invariant manner, the other at lower frequencies in a phase dependent manner. The (quasi)persistent, self-generating character of spiral waves is particularly interesting in the context of persistent neural responses to transient stimuli. Huang et al. (2010) suggests that spiral waves in visual cortex may be involved in maintaining persistent activity from transient stimuli in the sensory context. A video of their minimal computational model, in which a persistent spiral wave is seeded by transient input, is http://www.jneurosci.org/content/suppl/2004/11/03/24.44.9897.DC1/model-_spiraldrift.mpg. The current hypothesis extends this idea to the context of persistent activity in oculomotor postural control (Aksay et al., 2001; Major and Tank, 2004).

## 3. Fixational eye movements

Fixational eye movements can be quite different between species. Martinez-Conde and Macknik (2008) review comparative studies of FEM in different species, concluding that tremor appears to be the most phylogenetically conserved and fundamental component, consistent with a basic role for spiral wave dynamics in the generative process of FEM. Drift is also widespread, whilst microsaccades appear linked to the existence of foveated ocular architecture. Microsaccades are the most intensively researched component of FEM in humans. These fast relocations of the fixation point appear to play a similar role and manifest similar neural correlates as saccades more generally (Ko et al., 2010; Hafed and Krauzlis, 2012; Kagan and Hafed, 2013; Martinez-Conde et al., 2013; Otero-Millan et al., 2013; Poletti et al., 2013), but also show relations to drift (Engbert and Mergenthaler, 2006; Chen and Hafed, 2013). Microsaccades are relatively infrequent, occuring up to three times per second at most and usually less frequently, in an irregular but individually characteristic fashion (Engbert and Mergenthaler, 2006).

Most of fixation time (>90%) is occupied by a slow *drift* of fixation (Martinez-Conde et al., 2004; Martinez-Conde, 2006; Rolfs, 2009), as depicted in Figures 3, 4. Upon this is superimposed a fast (peaked around 80–90 Hz), low amplitude (approx. one photoreceptor), aperiodic oscillation termed *tremor*. Tremor is usually within the noise range of the recording equipment (Martinez-Conde et al., 2004). As a result, less is known about tremor than other components, and tremor is not resolved in many FEM studies of drift and microsaccades.

### 3.1. Dynamical characteristics of fixational eye movements

#### 3.1.1. Random walk modeling of FEM

On the basis of early studies (Cornsweet, 1956; Matin et al., 1970; Findlay, 1971), FEM have been widely held to exhibit the 1/*f* spectrum of classical Brownian noise. Random walk analysis examines the *mean squared displacement* of a diffusing “particle” (in this case the point of fixation) relative to time. In ideal Brownian motion, this relation is linear in time. This characterization has been adopted in some recent models of how vision may cope with (Pitkow et al., 2007; Burak et al., 2010), and indeed exploit (Kuang et al., 2012), jitter of the retinal image due to FEM.

At the motor end, an important neural correlate of oculomotor postural control is *persistent neural activity* in brainstem regions including the prepositus hypoglossi (“PH”) (Delgado-Garcia et al., 1989) and medial vestibular nucleus (“MVN”) (Serafin et al., 1991; du Lac and Lisberger, 1995). Persistent here refers to sustained activity on timescales much longer than individual neural spiking timescales, in response to a relatively brief stimulation. Seung (1996) described a model of how persistent neural activity could be maintained through positive feedback, and showed how FEM drift-tremor could reflect a random walk along the line attractor created by the positive feedback dynamics in the motor memory of eye position. Seung suggested that various sources of noise, such as the random fluctuations in the tonic input from vestibular afferents, could be causing the random walk behavior.

Persistent neural activity is associated with short term memory more generally (Major and Tank, 2004), and various potential mechanisms for maintaining persistent activity have been investigated (see for review Brody et al., 2003). Stability issues arising from the positive feedback model were addressed in Koulakov et al. (2002); Goldman et al. (2003). More recently, empirical evidence contrary to the predictions of line attractor models (Aksay et al., 2007; Miri et al., 2011a) has motivated the proposal of modifications of recurrant network models, and the development of new models based on functionally feedforward networks and balanced regimes of excitation and inhibition (Goldman, 2009; Lim and Goldman, 2013). We address this topic in more detail in the following section.

#### 3.1.2. Self-avoiding random walk models

Recent work has shown that FEM exhibit non-trivial temporal correlations whose description require fractional scaling exponents, rather than the unitary scaling exponent of pure Brownian motion. Engbert and Kliegl (2004); Mergenthaler and Engbert (2007) applied random walk analysis to the statistics of fixational eye movements at short (<40 ms) and long time (100–400 ms) scales. The tremor component was not resolved in these studies. At short timescales, the fixation point drifts faster than for normal diffusion/Brownian motion, with scaling exponents in the range 1.3–1.5. This is termed a “persistent” Codling et al. (2008) or “superdiffusive” Metzler and Klafter (2000) process. On longer timescales, however, the distance of the fixation point drifts slower than normal diffusion (termed subdiffusive/antipersistent). We adopt “sub/superdiffusive” here to avoid crossing terminology with that of persistent neural activity.

(Engbert et al., 2011) modeled this behavior in terms of a self-avoiding random walk (“SARW”) in a potential. The potential accounts for the long term subdiffusivity, implementing a tendency for “wandering back” to the center of the potential well in the long term. The short term superdiffusivity is modeled by giving the random walk a memory, and adding a term such that the walk next visits the neighbor whose “previously visited” activation is lowest, with ties resolved randomly. As a result, the walk tends to be structured by the initial direction which is chosen (randomly), as the path backwards is in general more visited. In this model, microsaccades are triggered by visiting a site with a “visited” activation above a threshold. Roberts et al. (2013) describe a similar self-avoiding random walk model, though without the confining potential and the microsaccade threshold, and with an imprecise continuous memory and rather than a lattice representation. Importantly, this study showed that both the superdiffusive drift in the FEM signal, and the self-avoiding random walk approach, generalize to more ecologically valid, dynamic viewing conditions (watching a film).

#### 3.1.3. Recent findings may challenge some existing ideas about FEM

Recently, Zhang and Li (2012) reported technical innovations based on binocular video tracking of ocular vein structure, which enable non-invasive, high resolution imaging of FEM in three rotational degrees of freedom. Importantly, the optical imaging approach avoids the interaction of mechanical measurement devices with ocular tremor. The authors reported a previously unsuspected level of structure at high resolution. See Figure 3 for a FEM trajectory where tremor is not resolved. Figure 4 depicts a FEM trajectory as measured by this new technique. These findings are quite new and have not received much attention to date, at least in terms of citations. They likely merit more attention, because if they are reproducible, they offer a challenge to existing conceptions of FEM in a number of ways.

Firstly, the microsaccadic trajectories recorded in these high resolution data are not straight and ballistic, as is widely supposed Martinez-Conde et al. (2004); Martinez-Conde (2006); Rolfs (2009); Martinez-Conde et al. (2013). The microsaccadic trajectories observed by Zhang and Li (2012) were often curved, and could exhibit fine scale changes in both speed and direction. Secondly, and crucially to our argument here, Zhang and Li (2012) reported that drift can take the form of a curled line at a fine spatial scale wherein tremor is resolved. This observation of a trajectory which is habitually self-crossing at a small spatiotemporal scale is not predicted by self-avoiding random walk models of drift generation, as the process should be self-avoiding at small spatiotemporal scales. These findings are a significant part of the motivation for the current hypothesis. Unfortunately, however, we have as yet been unable to obtain the associated time series data. Speculativeness notwithstanding, we believe that the proposal of testable hypotheses is a positive way to motivate the publicization of data and structure further empirical investigations in this area. Another high resolution non-contact FEM measurement method which can resolve tremor has recently been reported by Kenny et al. (2013a,b), so data of sufficient resolution may soon become available from this group.

#### 3.1.4. Tremor; a clue to the mechanisms of FEM, or “just noise”?

Tremor has often been dismissed as “noise,” but then so have other aspects of FEM over the years. Whether tremor reflects unrelated background noise or the workings of the neural mechanisms which maintain the dynamical posture of the eye remains very much an open question. Spauschus et al. (1999) found strong binocular coherence of tremor, and concluded that tremor reflects the patterning of low-level drives to oculomotor neurons, rather than motor noise. More recently, a sophisticated method was employed by Thiel et al. (2008), who reported positive evidence for binocular phase synchronization. They concluded that there might be only one center in the brain that produces the fixational movements in both eyes, or a close link between the two centers. The loss or reduction of tremor in certain cases of brain pathology (Michalik, 1987) and in coma (Shakhnovich and Thomas, 1977) also gives reason to suspect a more important, and delicate, source of tremor.

## 4. Motivations for the spiral wave hypothesis of FEM drift-tremor

### 4.1. Statistical similarities between drift-tremor trajectories and spiral dynamics

Tremor contains a strong spectral peak around 80–90 Hz and a less prominent, variable lower frequency component up to around 25 Hz (Spauschus et al., 1999). A low amplitude (approx. 1 photoreceptor), slow (around 5 Hz), tremor-like ocular oscillation has been recorded at very high resolution in the turtle (Greschner et al., 2002), possibly corresponding to the slow component of tremor in primates, though cross species comparisons must be made with caution (Martinez-Conde and Macknik, 2008). Greschner et al. (2002) reported this oscillation as periodic, though the flat peak in the frequency spectrum around 5 Hz, and the high variability visible in the inset example trajectories, may be suggestive of quasi-periodicity (see their Figure 1A). Either way, the regularity of this low frequency component is interesting because it suggests a certain systematicity to the generative mechanisms. Closer examination of human tremor is required to establish whether an identifiable carrier wave exists at lower frequencies, which is then heavily masked by the fast aperiodic component of tremor. An underlying spiral wave neurodynamics is consistent with both a periodic and a quasi-periodic form for this carrier wave (Barkley et al., 1990; Broer et al., 1996), and predicts the accompanying high frequency aperiodic oscillation (see Figures 1, 2).

See Figure 5, reprinted from Figure 7 in Huang et al. (2010), for visual representations of spiral drift recorded in visual cortex. Like FEM drift (Cornsweet, 1956; Matin et al., 1970; Findlay, 1971), spiral drift can exhibit Brownian statistical structure, due to both external forcing/environmental gradients (Sendiña-Nadal et al., 2000; Yuan et al., 2011) and intrinsic dynamics (Biktashev and Holden, 1998). Such gradients could (but need not) reflect the path memory and/or confining potentials in SARW models (Engbert et al., 2011), and perhaps even visual context (Mensh et al., 2004; Chan and Galiana, 2005). Like FEM drift, the velocity of spiral drift in neocortex is variable (Huang et al., 2010), and was found to be higher in induced sleep-like states (see Figure 5), consistent with recent observations that time-on-task increases the speed of FEM drift, whilst reducing the peak velocity of microsaccades (Di Stasi et al., 2013).

*Prediction* The singularity hypothesis predicts that, when the small spatial scale of tremor is resolved, drift-tremor trajectories will take a curling, self-crossing form reflecting the rotational orbit of a spiral wave. This might also be complex due to interactions between similar systems controlling different degrees of freedom. The fast component of tremor will manifest as an aperiodic modulation of this carrier wave. The presence of a slow (10–20 Hz) rotational component, giving the trajectory a habitually *self-crossing* form at small scales, would distinguish the spiral wave model from self-avoiding random walk models of FEM drift.

### 4.2. Spiral waves and the neurodynamics of oculomotor postural control

#### 4.2.1. Persistent neural activity

*Persistent neural activity* (“PNA”) refers to localized “bumps” of fast firing cells which persist over timescales much longer then the timescales of the individual neurons comprising the bump. PNA is an important neural correlate of working memory (Major and Tank, 2004), and has been studied intensively in the context of brainstem *neural integrators* which encode eye position during oculomotor control (e.g., Aksay et al., 2001, 2007; de Dios Navarro-López et al., 2004; Miri et al., 2011a). Fluctuations in oculomotor PNA are thought to underly FEM drift and tremor, but the precise mechanisms responsible for their generation remain a matter of considerable debate. Current models regarding these mechanisms have been categorized on the basis of whether they posit intrinsic unicellular mechanisms (e.g., Shen, 1989; Loewenstein and Sompolinsky, 2003; Teramae and Fukai, 2005) or network mechanisms (Cannon and Robinson, 1985; Seung, 1996; Seung et al., 2000; Goldman et al., 2003; Goldman, 2009). Some role for network mechanisms is suggested by evidence of correlated activity between cells in the oculomotor integrator network (Aksay et al., 2003), and by the observed covariance of eye position and the frequency and magnitude of the synaptic barrage converging on integrator cells (Huang, 2009). These alternatives are not necessarily mutually exclusive, and multiple mechanisms may operate in the maintainance of PNA in the oculomotor system and elsewhere in the brain (Major and Tank, 2004).

The vestibulo-oculomotor system exhibits fractional dynamics (Anastasio, 1994), and complex time variation in PNA has motivated arguments that a model with multiple timescales of persistent firing may be required (Anastasio, 1998). Indeed, recent evidence for multiple timescales of persistence in oculomotor PNA (Miri et al., 2011a) suggests a higher dimensional attractor dynamics than proposed by earlier line attractor models (Seung, 1996; Seung et al., 2000; Goldman et al., 2003), leading to the development of new models with more complex dynamics (Miri et al., 2011a; Fisher et al., 2013). (Goldman, 2009) describes a functionally feed-forward architecture which reproduces some of the time variation in PNA, showing that positive feedback is not essential in principle, while Lim and Goldman (2013) presents a model based on homeostatic mechanisms which maintain a careful balance of excitation and inhibition. In human psychophysical studies, Khojasteh et al. (2012) found that cross subject averaging hides idiosyncratic nonlinear patterns. All this suggests that considerable complexity inhabits the dynamics of PNA in the oculomotor system (Durstewitz and Seamans, 2006).

The current hypothesis suggests an addition to the repertoire of hypothesized mechanisms for PNA, which falls into the category of network mechanisms, though is distinct from existing network models in a number of ways. Unlike existing network models, which concentrate on modeling neural behavior at the level of firing rates and drift, we focus on the finer spatiotemporal scale of FEM tremor and subthreshold fluctuations in the membrane potential of cells mediating oculomotor integration. Rather than specific circuit design, persistence is based on the transient self-organization of population activity into a reentrant, (quasi)periodic spatiotemporal pattern. Spiral waves require a predominance of excitatory, spatially distributed connections but precise connectivity structure is not required; spirals can easily emerge in randomly connected networks (Milton et al., 1993; Chu et al., 1994; Yuan et al., 2011). This is not to say that specific circuitry is not important or present in the hVPNI; just that it is not a requirement of the current model. The existence of spatially organized, excitatory lateral connectivity is suggested by various studies (Aksay et al., 2001, 2007; Miri et al., 2011a). Disinhibition is crucial to the formation of spirals (and other traveling waves) in cortical tissue (Huang et al., 2004, 2010). (Aksay et al., 2007) identified that mutually inhibitory collateral interactions were not necessary to local integrators within a certain range, suggesting that these mutually inhibitory interactions regulated local mechanisms rather than driving PNA directly. Such a situation could relate to mutual modulation of the excitability required for the emergence and propagation of traveling waves.

#### 4.2.2. Functional properties of spiral waves

Certain features of spiral waves make them potentially interesting as neurodynamical mediators of PNA. Indeed, spiral waves are a form of neural activity which is persistent (e.g., Milton et al., 1993; Chu et al., 1994), though they are not usually associated with the term as used in the context of short term memory and neural integrators. The combination of A, B, C and D below suggests a mechanism capable of contributing to the maintenance of localized persistent neural activity.

*A. Spatiotemporally organized depolarization:* Sub-threshold traveling waves in the membrane potential field coordinate network activity in space and time, by defining spatiotemporal regimes of polarization-depolarization (Wu et al., 2008; Huang et al., 2010; Bahramisharif et al., 2013). Cells in the vicinity of the singularity can take arbitrarily differing phase, leading to an almost phaseless depolarizing synaptic barrage in that vicinity.

*B. Pseudo-locality:* Spiral waves exhibit a duality which gives them both a local, particle-like description (the singularity) and a global wave-like description (the propagating spiral arms) (Biktasheva and Biktashev, 2003). Though the wave is extensive, its behavior is almost entirely based on what happens in the neighborhood of the singularity.

*C. Quasi-persistence:* Spirals are reentrant waves, whose activity generates the conditions for their own persistence in time (Winfree, 1991), subject to certain conditions (e.g., Ito and Glass, 1991; Fenton et al., 2002; Zhang et al., 2003; Chun-Ni et al., 2010; Ma et al., 2010, 2012a). C combined with A and B, enables a spiral wave to *persistently* depolarize a *spatially localized* region in the neighborhood of the singularity.

*D. Seedability:* Spiral waves can be induced in an appropriate medium by various methods (Aranson et al., 1994; Williams and Holland, 1999; Leanhardt et al., 2002; Zhang et al., 2002; Xiao-Ping et al., 2011; Yuan et al., 2011; Ma et al., 2012b).

The most effective and tunable method is probably to directly impose an external forcing spiral, as in (Xiao-Ping et al., 2011), or a spiral seed plus a periodic forcing current near the singularity (Zhang et al., 2002). However, persistent spirals can also be induced in networks of integrate-and fire neurons by a brief, non-spiral periodic forcing (Milton et al., 1993; Chu et al., 1994; Huang et al., 2004; Kilpatrick and Bressloff, 2010b; Yuan et al., 2011) given some reasonable connectivity conditions (chiefly spatiality and some kind of inhomogeneity/noise/perturbation which breaks rotational symmetry).

D combined with A, B and C, provides a mechanism whereby an afferent may seed a spatial pattern in an efferent, and then leave that pattern to sustain itself with a certain amount of autonomy. Durstewitz and Deco (2008); Friston et al. (2012) suggest that brain activity is characterized by a high dimension chaotic background state, from which lower dimensional metastable states transiently emerge. Figure 6, from (Zhang et al., 2002) nicely visualizes the notion of how a spiral wave seeding might realize such a transient dimensionality reduction in the context of the observed (Aksay et al., 2001) difference between a background “off” state of the integrator, characterized by irregular firing at low rates (the turbulent background), and an “on” state characterized by driving input from the seeding of a spiral wave. de Dios Navarro-López et al. (2004) induced PNA in an oculomotor integrator circuit with brief, cholinergic periodic forcings. Oscillatory neurons observed in the guinea pig nucleus prepositus hypoglossi (Idoux et al., 2006), a region deeply associated with oculomotor integration (Delgado-Garcia et al., 1989; McCrea and Horn, 2006), might exemplify a neural substrate for periodic forcing inputs and the maintainance of traveling wave activity in the population. The persistent spiking of neurons in the vicinity of the induced singularity will be facilitated due to constant depolarization of the cellular membrane by high frequency microstimulation without a strong phasic component, as cells near the singularity may take arbitrarily different phase.

The bump of persistent firing activity in PNA has naturally been associated with “bumps” in neural field models (e.g., Tegnér et al., 2002; Owen et al., 2007; Kilpatrick and Ermentrout, 2013). However, it has also been suggested that hVPNI neurons may operate in a fluctuation dominated regime, in part because the firing threshold of the cell *increases with the membrane potential*, and in part because firing always occurs at the apex of membrane potential fluctuations (Huang, 2009). In a fluctuation dominated regime, firing rate is dependent less on mean membrane potential than on fast fluctuations in the level of the depolarizing synaptic barrage. On this view, at the level of subthreshold field dynamics fast microfluctuation (from a spiral wave) could be more effective than a constant raise in stimulation (from a bump) in inducing persistent firing. Thus spiral waves possess a unique repertoire of functional properties which render them interesting in the context of PNA. However, the presence or absence of a spiral wave only yields a binary distinction between an “on” state and an “off” state. Still missing is a mechanism for a graded temporal memory capable of remembering multiple, arbitrary step changes.

#### 4.2.3. Possible mechanisms for continuous temporal integration

Persistent neural activity is associated with *neural integration* (Major and Tank, 2004), and has been intensively studied in the anatomical context of oculomotor postural control, in particular the *horizontal velocity to position integrator* (“hVPNI”) (Aksay et al., 2000). Here, a cell integrates (in the mathematical sense) its inputs over time, providing the ability to hold, and externally nudge, set points. An external nudge is reflected in a persistent shift in average membrane potential, co-occuring with step changes in firing rate and eye position (Aksay et al., 2001). How might this graded integration functionality be implemented by a spiral wave depolarization regime?

One possibility here is that spiral waves of different spatial extent generate different levels of depolarizing input. Figure 6, reprinted from Zhang et al. (2002) depicts how different intensities of forcing current can generate different sizes of spiral wave, from a pre-existing background of low level, chaotic turbulence. Increasing the size of the spiral by adding energy could perhaps encode up steps, but it is less obvious how a down step would be implemented. Another possibility is inducing multiple spirals.

Functionally, however, modulation of the frequency of the spiral's rotational orbit could provide the most appropriate variable for graded temporal integration. The excitability of the medium has a strong determining effect on the frequency taken by spirals and other traveling waves (Winfree, 1991). Modulation of the strength of lateral connections might therefore provide a mechanism to induce persistent changes in spiral frequency. Calcium mediated presynaptic facilitation (Mongillo et al., 2008) could provide a mechanism for strengthening lateral connectivity, and other modes of disinhibition could also be relevant (e.g., de Dios Navarro-López et al., 2004). Kilpatrick and Bressloff (2010a,b) describe spirals in neural field models, in which spike frequency adaptation modulates the frequency of network oscillations. Whatever the mechanism of frequency modulation, a faster spiral would generate more action potentials per unit time in the synaptic barrage converging on the cell from weak but numerous lateral connections, maintaining membrane depolarization, and the magnitude of these would be amplified by lateral synaptic facilitation. Both the magnitude and the arrival frequency of depolarizing excitatory postsynaptic potentials converging on active eye position coding cells varies systematically with eye position Aksay et al. (2001); Huang (2009).

*Secondary Prediction* A spiral frequency based temporal integrator is consistent with the close covariance of the arrival frequency of action potentials with eye position (Huang, 2009), and would predict in addition that a spectral peak in the slower range (around 10–20 Hz) of membrane potential oscillations during PNA will vary systematically with eye position in *individual trial* data (averaging might hide this effect). Note that the frequency modulation approach to graded integration is a secondary hypothesis.

#### 4.2.4. Subthreshold dynamics of the membrane potential during PNA in an oculomotor integrator

Aksay et al. (2001) carried out *in vivo* intracellular recording and perturbation of persistent activity in an oculomotor neural integrator. They tracked the evolution of the cellular membrane potential during step changes in persistent activity associated with position control during fixation events. Their Figure 2 is reprinted here as Figure 7. See also their Figure 1 for longer recording period. Note the step like change in membrane potential that accompanies the onset of persistent firing. Further step changes are marked by brief (50–100 ms) overshoot/undershoot depending on direction of change, followed by a persistent change in the mean membrane potential and firing rate. The membrane depolarization was found to be sufficient to explain the associated PNA in a control experiment, suggesting an important role for network mechanisms.

Up (down) steps in membrane potential are correlated with increases (decreases) in both arrival frequency and magnitude of *excitatory postsynaptic potentials* (“EPSPs”) (Aksay et al., 2001; Huang, 2009). The membrane potential (*V*_*is*_)and the firing rate (*F*_intra_) shows signs of an oscillation at around 15 Hz, perhaps corresponding to the rotational orbit of a spiral wave and the slow component of tremor. If the barrage of depolarizing EPSPs are indeed originating from the slow rotational orbit and fast jitter of a spiral wave, then under close examination one would expect to see the leading edge of the spiral waveform reflected in the EPSPs. Huang (2009) examined membrane potential fluctuations during PNA in great detail. Whole-cell patch recordings revealed the existence of many small (0.2–3 mV) excitatory postsynaptic potentials lasting 5–10 ms, and manifesting a “peculiar” sharp-attack, slow-decay form obscured in accompanying sharp electrode recordings. See their section 5 and Figure 5.3, reproduced here as Figure 8. This waveform is typical of that generated by the passing of the leading edge of a spiral wave, where the phase gradient is very high (providing the sharp attack). See for example the depictions from Qu et al. (1999) of action potentials caused by cardiac spiral waves http://ajpheart.physiology.org/content/ajpheart/276/1/H269/F8.large.jpg. Note though that other possibilities exist. Based on similar waveforms observed in Mauthner cells (Golding and Spruston, 1998; Korn and Faber, 2005), Huang (2009) suggests that mixed NMDA/AMPA conductances could underly the shape of these potentials.

*Prediction* At the network level, the singularity hypothesis predicts that spiral waves should be directly identifiable in the VSDI signal at the site of PNA (at least in the anatomical context of brainstem oculomotor integration), and that the success (failure) to induce a spiral wave will distinguish success (failure) to induce PNA. While it is possible in principle that the membrane potential dynamics just reviewed could reflect a spiral wave which exists elsewhere, neurophysiological evidence suggests that the mechanisms sustaining PNA in the hVPNI are local (Aksay et al., 2007).

A standard test for whether an observation corresponds to a true spiral wave is the existence of a phase singularity, with a local amplitude reduction of field oscillations in the vicinity of the singularity (Winfree, 1991, 2001; Huang et al., 2010), caused by the cancellation of signals from closely located nodes with opposing phase near the singularity. See Figure 1A in Huang et al. (2010), reprinted here as Figure 9 for neurophysiological recordings of this phenomena. Small fluctations in the balance of this cancellation result in an increase in high frequency fluctuations visible in the VSDI signal in Figure 9 (and also visible as jitter of the singularity in our Figure 1), which could drive a cell effectively in a fluctuation dominated regime (Huang, 2009).

We are not aware of VSDI studies of brainstem neural integrators. Recent methodological advances may combine to provide opportunities for imaging the spatiotemporal dynamics of subthreshold activity in the deep brainstem. Fiber optics offer a means to image non-superficial regions (Flusberg et al., 2005). Combining VSDI and laser scanning microstimulation offers a fast method for anatomical and functional mapping (Xu et al., 2010). Zebra fish larvae have recently been shown to provide an *in vivo* preparation with high optical transparency (Miri et al., 2011a; Fisher et al., 2013). Miri et al. (2011b) used two-photon laser scanning microscopy (Stosiek et al., 2003) to simultaneously image many cells in neural integrator circuits in the larval zebra fish, and introduced a semi-automated approach for identifying behavior measure (in this case eye movement) related cells in the ensuing space-time series.

Combining this methodology with VSDI, which would provide access to subthreshold spatiotemporal dynamics associated with PNA, could test directly whether spiral waves exist and if so, whether they are spatially associated with active eye position integrators and whether their rotational frequency (and/or spatial extent) covaries systematically with eye position. Regardless of whether these predictions are confirmed or denied, VSDI data would likely to be of great utility in the general research effort on the hVPNI.

### 4.3. Saccades and microsaccades

The current contribution focuses on the dynamic maintainance of oculomotor posture between microsaccades. Nonetheless microsaccades, and indeed saccades in general, are naturally relevant to the discussion as a whole. In this section, we briefly address how a spiral wave model of drift-tremor might fit into its saccadic context.

#### 4.3.1. Scale free saccadic behaviors

Recent evidence points to a remarkable continuity in the statistics of saccadic oculomotor control across scales including microsaccades (Otero-Millan et al., 2013). How might a spiral wave model of fixational postural control fit into its containing context of fast (micro and macro) saccadic gaze shifts? Is there a continuously scaling control principle which could give rise to a visual scan path with, from the bottom up;

- microfixations characterized by spiral dynamics (drift-tremor), which are interspersed with
- relatively long, straight and fast flights (microsaccades), which are organized into
- clusters of microfixations (macrofixations), which are interspersed with
- relatively very long, straight, fast flights (macrosaccades), which in turn cluster into
- regions of dense exploration and short saccades, interspersed by long saccades to new regions of interest?

The natural variability of human scanning patterns has been modeled at the macro-saccadic level by the imposition of a stochastic component comprising a Lévy walk (Klafter et al., 1987) upon scanpaths in a deterministic salience landscape (Brockmann and Geisel, 1999; Boccignone and Ferraro, 2012). Another possibility is that the stochastic component reflects an intrinsic probabilistic feature of the salience function, (e.g., Harel et al., 2006), rather than an imposed randomization.

#### 4.3.2. Lévy walks in rotational and turbulent flow

Theoretical work has revealed deep links between spiral waves, turbulent flow, fractional Brownian motion, anomalous diffusion and Lévy type trajectories (Shlesinger et al., 1987; Viecelli, 1990; Metzler and Klafter, 2000). Solomon et al. (1993) observed Lévy walks of tracer particles in a physical system of effectively two dimensional rotating flow, and Solomon et al. (1994) examined in more detail behavior in periodic, chaotic and turbulent conditions. See our Figure 10 for a reprint of Figure 6 in Solomon et al. (1994), depicting example trajectories. Long term trajectories exhibited a pattern of long, relatively direct flights in predominantly translational flow, interspersed with episodes where the particle is caught up in a spiraling curve due to capture by a vortex (“sticking”). Analysis of sticking times and flight statistics indicated a Lévy walk trajectory evolving in continuous time. Biomechanical constraints suggest that a *truncated* Lévy walk Mantegna and Stanley (1994), where maximum step lengths are finite, may be more appropriate to the biological case.

Similar dynamics in a more complex landscape consisting of multiple clusters of vortices could conceivably result in a trajectory resembling that of multiscale visual exploration. “Fixation” periods consist of clusters of mini-fixations, each of which consists of an episode of vortex sticking characterized by rotational flow. Escaping a cluster results in a relatively long step to the next cluster (i.e., a macrosaccade), followed by a sequential sampling of the new cluster. The traveling wave accompanying saccadic execution observed in the superior colliculus by Munoz et al. (1991) might be a manifestation of relatively long flights between vortex sticking visible in Figure 10. The curling trajectory of drift-tremor during microfixation and the curving, interrupted microsaccade trajectories reported by Zhang and Li (2012) are reminiscent of the turbulent transport scenario just outlined, though closer analysis of these data is required. Note how the microsaccadic trajectories in Figure 4 do not always start their trajectory in the direction of their final destination. They are often curved and can have small scale variations in velocity. Looking closely at the microsaccades depicted, one may observe vertical motions exhibiting an oscillation which is damped in one horizontal dimension and amplified in the other, suggestive of transport in a potential field. Where they do travel in straight lines, this is usually on the horizontal axis, suggestive of a potential field. Overall the pattern is not ballistic, but is consistent with the trajectory first escaping local vortex sticking in an unpredictable direction, followed by a trajectory dominated by translational flow with potential fields. Figure 11 depicts an example of a trajectory in a model of anomalous transport in magnetic field turbulence, taken from Chiaravalloti et al. (2006). This provides an example, though from a different domain, of how these dynamics could generate trajectories similar to the FEM trajectories depicted in Figure 4. Adding a potential field might further approximate the FEM trajectories in Figure 4.

### 4.4. Turbulent transport and chaotic itinerancy

Thus there is some potential for a continuous, deterministic dynamical principle capable of generating the scale free, stochastic profile of visual scanning trajectories. This speculative proposed framework for multiscale visual exploration would imply a widespread role for traveling waves and rotational flow in brain-body hermeneutics, which may stretch the readers credulity at this stage, but there is some existing context. Breakspear (2001); Tyukin et al. (2009); Friston et al. (2012) examine traveling wave processing and self-organized instability in perception, whilst Heitmann (2013) explores traveling wave functionality in the motor context. In addition to noting a potential contribution to persistent neural activity in the sensory context, Huang et al. (2010) suggests that spiral waves, as a locally generated event, may also help a local cortical circuit to quickly disengage from globally synchronized rhythms. If traveling waves are playing functional roles in brain activity, one role of rotational flow may be to “hold the posture” of the central nervous system, while translational flow interconnects metastable postural transients. If so, this should be reflected in fixational drift-tremor and saccades, because the eye is part of the CNS. Breakspear et al. (2010) suggest that traveling wave solutions may offer optimal solutions to minimization of the free-energy in far from equilibrium initial conditions. Free energy minimization may be a rather general heuristic in nervous function (Friston, 2010). If the predictions of the singularity hypothesis of FEM drift-tremor turn out to be accurate, then the case for transient population dynamics as optimizers of behavior would gain a considerable boost.

It is interesting to speculate that attention may be related to nervous mechanisms of suppressing spiral waves. On this view, the sequential visiting of spirals in a cluster would destabilize the spirals and cause their breakup, resulting in a collateral effect resembling inhibition of return. This kind of self-destabilizing, itinerant trajectory would link action and perception into a common framework probably best described in terms of existing work on (embodied) *chaotic itinerancy* (Tsuda, 1991, 2009; Kaneko, 1992; Kaneko and Tsuda, 2003; Ikegami, 2007). Transient dynamics traversing a landscape of attractor ruins with riddled basins (Milnor, 1985) (i.e., quasi-attractors whose basin of attraction is riddled with repellent trajectories belonging to the basin of another attractor) can perform perceptual (Breakspear, 2001; Tyukin et al., 2009) and memory (Rabinovich et al., 2001) functions. This raises the possibility that the transient dynamics of embodied eye movements could play a rather sophisticated perceptual role analagous in computational description to that of neural sensory mechanisms (Tyukin et al., 2009), but at the embodied level where the perturbation structure of the world constitutes the data set (Ikegami, 2007). Wilkinson et al. (2011) give a simple computational example of how exploratory gaze patterns structured by spiral waves can enact perception of a global property of a social scene (co-orientation), as has been observed in infants (Augusti et al., 2010; Handl et al., 2013).

## 5. Conclusion

Despite extensive study of oculomotor postural control, the generative mechanisms of fixational drift and tremor remain uncertain. We have proposed the hypothesis that these components reflect the drift and meander of spiral wave neurodynamics. Whilst speculative, the singularity hypothesis offers a parsimonious and predictive account of FEM. Though our motivations are chiefly functional, the available psychophysical and neurophysiological evidence is largely consistent with, and occasionally suggestive of, a contribution of rotational flow to the maintainence of persistent neural activity in the oculomotor system. We have laid out an argument motivating our hypothesis in terms of the existing literature, and made testable predictions which could falsify it. Our hope is that these predictions will encourage other groups working in the fields of FEM and oculomotor integration to consider looking for indicators of rotational flow when analysing data, and perhaps even motivate neuroimaging studies to examine the subthreshold spatiotemporal dynamics associated with PNA. Should empirical studies and/or analysis of existing data confirm the basic predictions of the conceptual model, future work should undertake detailed computational modeling. Further testing and development may offer a deterministic account of the stochasticity and self-similarity manifest in eye movement patterns across scales, based on the complex dynamics of anomalous transport in rotating neural flow.

### Conflict of interest statement

The authors declare that the research was conducted in the absence of any commercial or financial relationships that could be construed as a potential conflict of interest.

## References

[B1] Ahissar E. Arieli A. (2001). Figuring space by time. Neuron 32, 185–201 10.1016/S0896-6273(01)00466-4 11683990

[B2] Ahissar E. Arieli A. (2012). Seeing via miniature eye movements: a dynamic hypothesis for vision. Front. Comput. Neurosci. 6:89 10.3389/fncom.2012.00089 PMC3492788 23162458

[B3] Aksay E. Baker R. Seung H. Tank D. (2000). Anatomy and discharge properties of pre-motor neurons in the goldfish medulla that have eye-position signals during fixations. J. Neurophysiol. 84, 1035–1049 10938326 10.1152/jn.2000.84.2.1035

[B4] Aksay E. Baker R. Seung H. S. Tank D. W. (2003). Correlated discharge among cell pairs within the oculomotor horizontal velocity-to-position integrator. J. Neurosci. 23, 10852–10858 14645478 10.1523/JNEUROSCI.23-34-10852.2003 PMC6740981

[B5] Aksay E. Gamkrelidze G. Seung H. Baker R. Tank D. (2001). *In vivo* intracellular recording and perturbation of persistent activity in a neural integrator. Nat. Neurosci. 4, 184–193 10.1038/84023 11175880

[B6] Aksay E. Olasagasti I. Mensh B. D. Baker R. Goldman M. S. Tank D. W. (2007). Functional dissection of circuitry in a neural integrator. Nat. Neurosci. 10, 494–504 10.1038/nn1877 17369822 PMC2803116

[B7] Amit D. J. (1992). Modeling Brain Function: The World of Attractor Neural Networks. Cambridge: Cambridge University Press

[B8] Anastasio T. J. (1994). The fractional-order dynamics of brainstem vestibulo-oculomotor neurons. Biol. Cybern. 72, 69–79 10.1007/BF00206239 7880915

[B9] Anastasio T. J. (1998). Nonuniformity in the linear network model of the oculomotor integrator produces approximately fractional-order dynamics and more realistic neuron behavior. Biol. Cybern. 79, 377–391 10.1007/s004220050487 9851019

[B10] Aranson I. Levine H. Tsimring L. (1994). Controlling spatiotemporal chaos. Phys. Rev. Lett. 72:2561 10.1103/PhysRevLett.72.2561 10055915

[B11] Aston-Jones G. Cohen J. D. (2005). An integrative theory of locus coeruleus-norepinephrine function: adaptive gain and optimal performance. Annu. Rev. Neurosci. 28, 403–450 10.1146/annurev.neuro.28.061604.135709 16022602

[B12] Augusti E.-M. Melinder A. Gredebäck G. (2010). Look who's talking: pre-verbal infants perception of face-to-face and back-to-back social interactions. Front. Psychol. 1:161 10.3389/fpsyg.2010.00161 21833226 PMC3153775

[B13] Bahramisharif A. van Gerven M. A. Aarnoutse E. J. Mercier M. R. Schwartz T. H. Foxe J. J. (2013). Propagating neocortical gamma bursts are coordinated by traveling alpha waves. J. Neurosci. 33, 18849–18854 10.1523/JNEUROSCI.2455-13.2013 24285891 PMC4262700

[B14] Bak P. (1996). How nature works: the science of self-organized criticality. Nature 383, 772–773 10193053

[B15] Bak P. Tang C. Wiesenfeld K. (1987). Self-organized criticality: an explanation of 1/f noise. Phys. Rev. Lett. 59, 381–384 10.1103/PhysRevLett.59.381 10035754

[B16] Barkley D. Kness M. Tuckerman L. S. (1990). Spiral-wave dynamics in a simple model of excitable media: the transition from simple to compound rotation. Phys. Rev. A 42, 2489–2492 10.1103/PhysRevA.42.2489 9904313

[B17] Biktashev V. Holden A. (1993). Resonant drift of an autowave vortex in a bounded medium. Phys. Lett. A 181, 216–224 10.1016/0375-9601(93)90642-D

[B18] Biktashev V. Holden A. (1995). Resonant drift of autowave vortices in two dimensions and the effects of boundaries and inhomogeneities. Chaos Solit. Fract. 5, 575–622 10.1016/0960-0779(93)E0044-C

[B19] Biktashev V. Holden A. (1998). Deterministic brownian motion in the hypermeander of spiral waves. Physica D 116, 342–354 10.1016/S0167-2789(97)00304-7

[B20] Biktashev V. N. (2007). Drift of spiral waves. Scholarpedia 2:1836 10.4249/scholarpedia.1836

[B21] Biktasheva I. Biktashev V. (2003). Wave-particle dualism of spiral waves dynamics. Phys. Rev. E 67:026221 10.1103/PhysRevE.67.026221 12636790

[B22] Boccignone G. Ferraro M. (2012). Gaze shift behavior on video as composite information foraging. Sig. Process. Image Commun. 28, 949–966 10.1016/j.image.2012.07.002

[B23] Boerlijst M. C. Hogeweg P. (1991). Spiral wave structure in pre-biotic evolution: hypercycles stable against parasites. Physica D 48, 17–28 10.1016/0167-2789(91)90049-F

[B24] Bray M.-A. Lin S.-F. Aliev R. R. Roth B. J. Wikswo J. P. (2001). Experimental and theoretical analysis of phase singularity dynamics in cardiac tissue. J. Cardiovasc. Electrophysiol. 12, 716–722 10.1046/j.1540-8167.2001.00716.x 11405407

[B25] Breakspear M. (2001). Perception of odors by a nonlinear model of the olfactory bulb. Int. J. Neural Syst. 11, 101–124 10.1142/S0129065701000564 14632166

[B26] Breakspear M. Heitmann S. Daffertshofer A. (2010). Generative models of cortical oscillations: neurobiological implications of the kuramoto model. Front. Hum. Neurosci. 4:190 10.3389/fnhum.2010.00190 21151358 PMC2995481

[B27] Bressloff P. C. Cowan J. D. Golubitsky M. Thomas P. J. Wiener M. C. (2001). Geometric visual hallucinations, euclidean symmetry and the functional architecture of striate cortex. Philos. Trans. R. Soc. Lond. B Biol. Sci. 356, 299–330 10.1098/rstb.2000.0769 11316482 PMC1088430

[B28] Brockmann D. Geisel T. (1999). Are human scanpaths levy flights?, in Artificial Neural Networks, 1999. ICANN 99. Ninth International Conference on (Conf. Publ. No. 470), Vol. 1 (Edinburgh), 263–268

[B29] Brody C. D. Romo R. Kepecs A. (2003). Basic mechanisms for graded persistent activity: discrete attractors, continuous attractors, and dynamic representations. Curr. Opin. Neurobiol. 13, 204–211 10.1016/S0959-4388(03)00050-3 12744975

[B30] Broer H. W. Huitema G. B. Sevryuk M. B. (1996). Quasi-periodic motions in families of dynamical systems: order amidst chaos. Berlin: Springer

[B31] Burak Y. Rokni U. Meister M. Sompolinsky H. (2010). Bayesian model of dynamic image stabilization in the visual system. Proc. Natl. Acad. Sci. U.S.A. 107, 19525–19530 10.1073/pnas.1006076107 20937893 PMC2984143

[B32] Cannon S. C. Robinson D. A. (1985). An improved neural-network model for the neural integrator of the oculomotor system: more realistic neuron behavior. Biol. Cybern. 53, 93–108 10.1007/BF00337026 4084616

[B33] Chan W. Galiana H. L. (2005). Integrator function in the oculomotor system is dependent on sensory context. J. Neurophysiol. 93, 3709–3717 10.1152/jn.00814.2004 15703232

[B34] Chen C.-Y. Hafed Z. M. (2013). Postmicrosaccadic enhancement of slow eye movements. J. Neurosci. 33, 5375–5386 10.1523/JNEUROSCI.3703-12.2013 23516303 PMC6704992

[B35] Chiaravalloti F. Milovanov A. V. Zimbardo G. (2006). Self-similar transport processes in a two-dimensional realization of multiscale magnetic field turbulence. Phys. Scr. 2006:79 10.1088/0031-8949/2006/T122/012

[B36] Chu P. Milton J. G. Cowan J. D. (1994). Connectivity and the dynamics of integrate-and-fire neural networks. Int. J. Bifurcat. Chaos 4, 237–237 10.1142/S0218127494000198

[B37] Chun-Ni W. Jun M. Jun T. Yan-Long L. (2010). Instability and death of spiral wave in a two-dimensional array of hindmarsh–rose neurons. Commun. Theor. Phys. 53:382 10.1088/0253-6102/53/2/32

[B38] Codling E. A. Plank M. J. Benhamou S. (2008). Random walk models in biology. J. R. Soc. Interface 5, 813–834 10.1098/rsif.2008.0014 18426776 PMC2504494

[B39] Coombes S. (2005). Waves, bumps, and patterns in neural field theories. Biol. Cybern. 93, 91–108 10.1007/s00422-005-0574-y 16059785

[B40] Cornell-Bell A. Finkbeiner S. (1991). Ca^2+^ waves in astrocytes. Cell Calcium 12, 185–204 10.1016/0143-4160(91)90020-F 1647876

[B41] Cornsweet T. N. (1956). Determination of the stimuli for involuntary drifts and saccadic eye movements. JOSA 46, 987–988 10.1364/JOSA.46.000987 13367941

[B42] Cross M. C. Hohenberg P. C. (1993). Pattern formation outside of equilibrium. Rev. Mod. Phys. 65:851 10.1103/RevModPhys.65.851

[B43] Da-sheng L. S.-K. Y. (1980). The spiral structure of the tropical cyclone. Acta Meteorologica Sin. 3

[B44] de Dios Navarro-López J. Alvarado J. C. Márquez-Ruiz J. Escudero M. Delgado-García J. M. Yajeya J. (2004). A cholinergic synaptically triggered event participates in the generation of persistent activity necessary for eye fixation. J. Neurosci. 24, 5109–5118 10.1523/JNEUROSCI.0235-04.2004 15175380 PMC6729203

[B45] Delgado-Garcia J. Vidal P. Gomez C. Berthoz A. (1989). A neurophysiological study of prepositus hypoglossi neurons projecting to oculomotor and preoculomotor nuclei in the alert cat. Neuroscience 29, 291–307 10.1016/0306-4522(89)90058-4 2725860

[B46] Di Stasi L. L. McCamy M. B. Catena A. Macknik S. L. Cañas J. J. Martinez-Conde S. (2013). Microsaccade and drift dynamics reflect mental fatigue. Eur. J. Neurosci. 38, 2389–2398 10.1111/ejn.12248 23675850

[B47] du Lac S. Lisberger S. G. (1995). Cellular processing of temporal information in medial vestibular nucleus neurons. J. Neurosci. 15, 8000–8010 8613737 10.1523/JNEUROSCI.15-12-08000.1995 PMC6577971

[B48] Durstewitz D. Deco G. (2008). Computational significance of transient dynamics in cortical networks. Eur. J. Neurosci. 27, 217–227 10.1111/j.1460-9568.2007.05976.x 18093174

[B49] Durstewitz D. Seamans J. (2006). Beyond bistability: biophysics and temporal dynamics of working memory. Neuroscience 139, 119–133 10.1016/j.neuroscience.2005.06.094 16326020

[B50] Engbert R. Kliegl R. (2004). Microsaccades keep the eyes' balance during fixation. Psychol. Sci. 15, 431–431 10.1111/j.0956-7976.2004.00697.x 15147499

[B51] Engbert R. Mergenthaler K. (2006). Microsaccades are triggered by low retinal image slip. Proc. Natl. Acad. Sci. U.S.A. 103, 7192–7197 10.1073/pnas.0509557103 16632611 PMC1459039

[B52] Engbert R. Mergenthaler K. Sinn P. Pikovsky A. (2011). An integrated model of fixational eye movements and microsaccades. Proc. Natl. Acad. Sci. U.S.A. 108, E765–E770 10.1073/pnas.1102730108 21873243 PMC3182695

[B53] Ermentrout B. (1998). Neural networks as spatio-temporal pattern-forming systems. Rep. prog. Phys. 61:353 10.1088/0034-4885/61/4/002

[B54] Fenton F. H. Cherry E. M. Hastings H. M. Evans S. J. (2002). Multiple mechanisms of spiral wave breakup in a model of cardiac electrical activity. Chaos 12, 852–892 10.1063/1.1504242 12779613

[B55] Findlay J. (1971). Frequency analysis of human involuntary eye movement. Kybernetik 8, 207–214 10.1007/BF00288749 5090383

[B56] Finkbeiner S. (1992). Calcium waves in astrocytes-filling in the gaps. Neuron 8, 1101–1108 10.1016/0896-6273(92)90131-V 1351732

[B57] Fisher D. Olasagasti I. Tank D. W. Aksay E. R. Goldman M. S. (2013). A modeling framework for deriving the structural and functional architecture of a short-term memory microcircuit. Neuron 79, 987–1000 10.1016/j.neuron.2013.06.041 24012010 PMC3768012

[B58] Flusberg B. A. Cocker E. D. Piyawattanametha W. Jung J. C. Cheung E. L. Schnitzer M. J. (2005). Fiber-optic fluorescence imaging. Nat. Methods 2, 941–950 10.1038/nmeth820 16299479 PMC2849801

[B59] Freeman W. J. (2009). Vortices in brain activity: their mechanism and significance for perception. Neural Netw. 22, 491–501 10.1016/j.neunet.2009.06.050 19625165

[B60] Friston K. (2010). The free-energy principle: a unified brain theory? Nat. Rev. Neurosci. 11, 127–138 10.1038/nrn2787 20068583

[B61] Friston K. Breakspear M. Deco G. (2012). Perception and self-organized instability. Front. Computat. Neurosci. 6:44 10.3389/fncom.2012.00044 22783185 PMC3390798

[B62] Froese T. Woodward A. Ikegami T. (2013). Turing instabilities in biology, culture, and consciousness? On the enactive origins of symbolic material culture. Adapt. Behav. 21, 199–214 10.1177/1059712313483145

[B63] Goldberger A. L. West B. J. (1987). Fractals in physiology and medicine. Yale J. Biol. Med. 60, 421 3424875 PMC2590346

[B64] Golding N. L. Spruston N. (1998). Dendritic sodium spikes are variable triggers of axonal action potentials in hippocampal ca1 pyramidal neurons. Neuron 21, 1189–1200 10.1016/S0896-6273(00)80635-2 9856473

[B65] Goldman M. S. (2009). Memory without feedback in a neural network. Neuron 61, 621–634 10.1016/j.neuron.2008.12.012 19249281 PMC2674525

[B66] Goldman M. S. Levine J. H. Major G. Tank D. W. Seung H. (2003). Robust persistent neural activity in a model integrator with multiple hysteretic dendrites per neuron. Cereb. Cortex 13, 1185–1195 10.1093/cercor/bhg095 14576210

[B67] Gray R. A. Chattipakorn N. (2005). Termination of spiral waves during cardiac fibrillation via shock-induced phase resetting. Proc. Natl. Acad. Sci. U.S.A. 102, 4672–4677 10.1073/pnas.0407860102 15769861 PMC555701

[B68] Gray R. A. Jalife J. (1996). Spiral waves and the heart. Int. J. Bifurcat. Chaos 6, 415–435 10.1142/S0218127496000163

[B69] Gray R. A. Pertsov A. M. Jalife J. (1998). Spatial and temporal organization during cardiac fibrillation. Nature 392, 75–78 10.1038/32164 9510249

[B70] Greschner M. Bongard M. Rujan P. Ammermüller J. (2002). Retinal ganglion cell synchronization by fixational eye movements improves feature estimation. Nat. Neurosci. 5, 341–347 10.1038/nn821 11914721

[B71] Grinvald A. Hildesheim R. (2004). Vsdi: a new era in functional imaging of cortical dynamics. Nat. Rev. Neurosci. 5, 874–885 10.1038/nrn1536 15496865

[B72] Hafed Z. M. Krauzlis R. J. (2012). Similarity of superior colliculus involvement in microsaccade and saccade generation. J. Neurophysiol. 107, 1904–1916 10.1152/jn.01125.2011 22236714 PMC3331665

[B73] Handl A. Mahlberg T. Norling S. Gredebäck G. (2013). Facing still faces: what visual cues affect infants observations of others? Infant Behav. Dev. 36, 583–586 10.1016/j.infbeh.2013.06.001 23831615

[B74] Harel J. Koch C. Perona P. (2006). Graph-based visual saliency. Adv. Neural Inform. Process. Syst. 19, 545–552 24427198

[B75] Heitmann S. A. (2013). Principles of Encoding Motor Commands in Travelling Waves of Neural Oscillations. PhD thesis, University of New South wales

[B76] Heitmann S. Boonstra T. Breakspear M. (2013). A dendritic mechanism for decoding traveling waves: principles and applications to motor cortex. PLoS Comput. Biol. 9:e1003260 10.1371/journal.pcbi.1003260 24204220 PMC3814333

[B77] Heitmann S. Gong P. Breakspear M. (2012). A computational role for bistability and traveling waves in motor cortex. Front. Comput. Neurosci. 6:67 10.3389/fncom.2012.00067 PMC3438483 22973223

[B78] Hillyard S. A. Vogel E. K. Luck S. J. (1998). Sensory gain control (amplification) as a mechanism of selective attention: electrophysiological and neuroimaging evidence. Philos. Trans. R. Soc. Lond. B Biol. Sci. 353, 1257–1270 10.1098/rstb.1998.0281 9770220 PMC1692341

[B79] Huang X. Troy W. C. Yang Q. Ma H. Laing C. R. Schiff S. J. (2004). Spiral waves in disinhibited mammalian neocortex. J. Neurosci. 24, 9897–9902 10.1523/JNEUROSCI.2705-04.2004 15525774 PMC4413915

[B80] Huang X. Xu W. Liang J. Takagaki K. Gao X. Wu J.-Y. (2010). Spiral wave dynamics in neocortex. Neuron 68, 978–990 10.1016/j.neuron.2010.11.007 PMC4433058 21145009

[B81] Huang Z. (2009). Membrane potential fluctuations in a neural integrator. PhD thesis, Princeton University

[B82] Idoux E. Serafin M. Fort P. Vidal P.-P. Beraneck M. Vibert N. (2006). Oscillatory and intrinsic membrane properties of guinea pig nucleus prepositus hypoglossi neurons *in vitro*. J. Neurophysiol. 96, 175–196 10.1152/jn.01355.2005 16598060

[B83] Ikegami T. (2007). Simulating active perception and mental imagery with embodied chaotic itinerancy. J. Conscious. Stud. 14, 111–125

[B84] Ito H. Glass L. (1991). Spiral breakup in a new model of discrete excitable media. Phys. Rev. Lett. 66, 671 10.1103/PhysRevLett.66.671 10043869

[B85] Jung P. Cornell-Bell A. Madden K. S. Moss F. (1998). Noise-induced spiral waves in astrocyte syncytia show evidence of self-organized criticality. J. Neurophysiol. 79, 1098–1101 9463465 10.1152/jn.1998.79.2.1098

[B86] Kagan I. Hafed Z. M. (2013). Active vision: microsaccades direct the eye to where it matters most. Curr. Biol. 23, R712–R714 10.1016/j.cub.2013.07.038 24028947

[B87] Kaneko K. (1992). Overview of coupled map lattices. Chaos 2, 279 10.1063/1.165869 12779975

[B88] Kaneko K. Tsuda I. (2003). Chaotic itinerancy. Chaos 13, 926–936 10.1063/1.1607783 12946185

[B89] Kelso J. (1995). Dynamic Patterns: The Self Organization of Brain and Behaviour. Cambridge, MA: The MIT Press

[B90] Kenny E. Coakley D. Boyle G. (2013a). Biospeckle in the human sclera and impact on laser speckle correlation measurement of eye tremor. J. Biomed. Opt. 18, 097009–097009 10.1117/1.JBO.18.9.097009

[B91] Kenny E. Coakley D. Boyle G. (2013b). Ocular microtremor measurement using laser-speckle metrology. J. Biomed. Opt. 18, 016010–016010 10.1117/1.JBO.18.1.016010 23306826

[B92] Khojasteh E. Bockisch C. J. Straumann D. Hegemann S. C. (2012). A re-examination of the time constant of the oculomotor neural integrator in human, in Engineering in Medicine and Biology Society (EMBC), 2012 Annual International Conference of the IEEE (Osaka), 4780–4783 10.1109/EMBC.2012.6347036 23366997

[B93] Kilpatrick Z. P. Bressloff P. C. (2010a). Effects of synaptic depression and adaptation on spatiotemporal dynamics of an excitatory neuronal network. Physica D 239, 547–560 10.1016/j.physd.2009.06.003

[B94] Kilpatrick Z. P. Bressloff P. C. (2010b). Spatially structured oscillations in a two-dimensional excitatory neuronal network with synaptic depression. J. Comput. Neurosci. 28, 193–209 10.1007/s10827-009-0199-6 19866351

[B95] Kilpatrick Z. P. Ermentrout B. (2012a). Response of traveling waves to transient inputs in neural fields. Phys. Rev. E 85:021910 10.1103/PhysRevE.85.021910 22463247

[B96] Kilpatrick Z. P. Ermentrout B. (2013). Wandering bumps in stochastic neural fields. SIAM J. Appl. Dyn. Syst. 12, 61–94 10.1137/120877106 PMC10798676 38250343

[B97] Kilpatrick Z. P. Ermentrout G. B. (2012b). Hallucinogen persisting perception disorder in neuronal networks with adaptation. J. Comput. Neurosci. 32, 25–53 10.1007/s10827-011-0335-y 21671074

[B98] Klafter J. Blumen A. Shlesinger M. F. (1987). Stochastic pathway to anomalous diffusion. Phys. Rev. A 35:3081 10.1103/PhysRevA.35.3081 9898509

[B99] Ko H.-K. Poletti M. Rucci M. (2010). Microsaccades precisely relocate gaze in a high visual acuity task. Nat. Neurosci. 13, 1549–1553 10.1038/nn.2663 21037583 PMC3058801

[B100] Korn H. Faber D. S. (2005). The mauthner cell half a century later: a neurobiological model for decision-making? Neuron 47, 13–28 10.1016/j.neuron.2005.05.019 15996545

[B101] Koulakov A. A. Raghavachari S. Kepecs A. Lisman J. E. (2002). Model for a robust neural integrator. Nat. Neurosci. 5, 775–782 10.1038/nn893 12134153

[B102] Kuang X. Poletti M. Victor J. D. Rucci M. (2012). Temporal encoding of spatial information during active visual fixation. Curr. Biol. 22, 510–514 10.1016/j.cub.2012.01.050 22342751 PMC3332095

[B103] Kuramoto Y. Koga S. (1981). Turbulized rotating chemical waves. Prog. Theor. Phys. 66, 1081–1085 10.1143/PTP.66.1081

[B104] Langham J. Barkley D. (2013). Non-specular reflections in a macroscopic system with wave-particle duality: Spiral waves in bounded media. Chaos 23, 013134–013134 10.1063/1.4793783 23556971

[B105] Leanhardt A. Görlitz A. Chikkatur A. Kielpinski D. Shin Y. Pritchard D. (2002). Imprinting vortices in a bose-einstein condensate using topological phases. Phys. Rev. Lett. 89:190403 10.1103/PhysRevLett.89.190403 12443104

[B106] Lechleiter J. Girard S. Peralta E. Clapham D. (1991). Spiral calcium wave propagation and annihilation in xenopus laevis oocytes. Science 252, 123–126 10.1126/science.2011747 2011747

[B107] Li J. Zhang X. (2012). Using high-speed photography and image processing for fixational eye movements measurement, in Imaging Systems and Techniques (IST), 2012 IEEE International Conference on (Manchester), 28–33

[B108] Lim S. Goldman M. S. (2013). Balanced cortical microcircuitry for maintaining information in working memory. Nat. Neurosci. 16, 1306–1314 10.1038/nn.3492 23955560 PMC3772089

[B109] Loewenstein Y. Sompolinsky H. (2003). Temporal integration by calcium dynamics in a model neuron. Nat. Neurosci. 6, 961–967 10.1038/nn1109 12937421

[B110] Ma J. Huang L. Tang J. Ying H.-P. Jin W.-Y. (2012a). Spiral wave death, breakup induced by ion channel poisoning on regular hodgkin–huxley neuronal networks. Commun. Nonlin. Sci. Numer. Simul. 17, 4281–4293 10.1016/j.cnsns.2012.03.009

[B111] Ma J. Liu Q. Ying H. Wu Y. (2012b). Emergence of spiral wave induced by defects block. Commun. Nonlin. Sci. Numer. Simul. 18, 1665–1675 10.1016/j.cnsns.2012.11.016

[B112] Ma J. Tang J. Zhang A. Jia Y. (2010). Robustness and breakup of the spiral wave in a two-dimensional lattice network of neurons. Sci. China Phys. Mech. Astron. 53, 672–679 10.1007/s11433-010-0097-y

[B113] Maass W. Natschläger T. Markram H. (2002). Real-time computing without stable states: a new framework for neural computation based on perturbations. Neural Comput. 14, 2531–2560 10.1162/089976602760407955 12433288

[B114] Major G. Tank D. (2004). Persistent neural activity: prevalence and mechanisms. Curr. Opin. Neurobiol. 14, 675–684 10.1016/j.conb.2004.10.017 15582368

[B115] Mantegna R. N. Stanley H. E. (1994). Stochastic process with ultraslow convergence to a gaussian: the truncated lévy flight. Phys. Rev. Lett. 73:2946 10.1103/PhysRevLett.73.2946 10057243

[B116] Matin L. Matin E. Pearce D. G. (1970). Eye movements in the dark during the attempt to maintain a prior fixation position. Vis. Res. 10, 837–857 10.1016/0042-6989(70)90164-1 5492775

[B117] Martinez-Conde S. (2006). Fixational eye movements in normal and pathological vision. Prog. Brain Res. 154, 151–176 10.1016/S0079-6123(06)54008-7 17010709

[B118] Martinez-Conde S. Macknik S. L. (2008). Fixational eye movements across vertebrates: comparative dynamics, physiology, and perception. J. Vis. 8:28 10.1167/8.14.28 19146329

[B119] Martinez-Conde S. Macknik S. L. Hubel D. H. (2004). The role of fixational eye movements in visual perception. Nat. Rev. Neurosci. 5, 229–240 10.1038/nrn1348 14976522

[B120] Martinez-Conde S. Otero-Millan J. Macknik S. L. (2013). The impact of microsaccades on vision: towards a unified theory of saccadic function. Nat. Rev. Neurosci. 14, 83–96 10.1038/nrn3405 23329159

[B121] McCrea R. A. Horn A. K. (2006). Nucleus prepositus. Prog. Brain Res. 151, 205–230 10.1016/S0079-6123(05)51007-0 16221590

[B122] Mensh B. Aksay E. Lee D. Seung H. Tank D. (2004). Spontaneous eye movements in goldfish: oculomotor integrator performance, plasticity, and dependence on visual feedback. Vis. Res. 44, 711–726 10.1016/j.visres.2003.10.015 14751555

[B123] Mergenthaler K. Engbert R. (2007). Modeling the control of fixational eye movements with neurophysiological delays. Phys. Rev. Lett. 98:138104 10.1103/PhysRevLett.98.138104 17501244

[B124] Metzler R. Klafter J. (2000). The random walk's guide to anomalous diffusion: a fractional dynamics approach. Phys. Rep. 339, 1–77 10.1016/S0370-1573(00)00070-3

[B125] Michalik M. (1987). Spektralanalysen des okulären mikrotremors bei hirnstammfunktionsstörungen. EEG. EMG. Z. Elektroenzephalogr. Elektromyogr. Verwandte Geb. 18, 20–26 3106001

[B126] Milnor J. (1985). On the concept of attractor. Commun. Math. Phys. 99, 177–195

[B127] Milton J. Jung P. (2003). Epilepsy as a Dynamic Disease. Berlin: Springer 10.1007/978-3-662-05048-4

[B128] Milton J. G. (2012). Neuronal avalanches, epileptic quakes and other transient forms of neurodynamics. Eur. J. Neurosci. 36, 2156–2163 10.1111/j.1460-9568.2012.08102.x 22805061

[B129] Milton J. G. Chu P. H. Cowan J. D. (1993). Spiral waves in integrate-and-fire neural networks. Adv. Neural Inform. Process. Syst. 5, 1001–1006

[B130] Miri A. Daie K. Arrenberg A. B. Baier H. Aksay E. Tank D. W. (2011a). Spatial gradients and multidimensional dynamics in a neural integrator circuit. Nat. Neurosci. 14, 1150–1159 10.1038/nn.2888 21857656 PMC3624014

[B131] Miri A. Daie K. Burdine R. D. Aksay E. Tank D. W. (2011b). Regression-based identification of behavior-encoding neurons during large-scale optical imaging of neural activity at cellular resolution. J. Neurophysiol. 105, 964–980 10.1152/jn.00702.2010 21084686 PMC3059183

[B132] Modolo J. Legros A. Thomas A. W. Beuter A. (2011). Model-driven therapeutic treatment of neurological disorders: reshaping brain rhythms with neuromodulation. Interface Focus 1, 61–74 10.1098/rsfs.2010.0509 22419974 PMC3262241

[B133] Molina-Terriza G. Torres J. P. Torner L. (2007). Twisted photons. Nat. Phys. 3, 305–310 10.1038/nphys607

[B134] Mongillo G. Barak O. Tsodyks M. (2008). Synaptic theory of working memory. Science 319, 1543–1546 10.1126/science.1150769 18339943

[B135] Munoz D. P. Pelisson D. Guitton D. (1991). Movement of neural activity on the superior colliculus motor map during gaze shifts. Science 251, 1358–1360 10.1126/science.2003221 2003221

[B136] Olsen S. R. Bortone D. S. Adesnik H. Scanziani M. (2012). Gain control by layer six in cortical circuits of vision. Nature 483, 47–52 10.1038/nature10835 22367547 PMC3636977

[B137] O'Regan J. K. Noë A. (2001). A sensorimotor account of vision and visual consciousness. Behav. Brain Sci. 24, 939–972 10.1017/S0140525X01000115 12239892

[B138] Otero-Millan J. Macknik S. L. Langston R. E. Martinez-Conde S. (2013). An oculomotor continuum from exploration to fixation. Proc. Natl. Acad. Sci. U.S.A. 110, 6175–6180 10.1073/pnas.1222715110 PMC3625326 23533278

[B139] Owen M. Laing C. Coombes S. (2007). Bumps and rings in a two-dimensional neural field: splitting and rotational instabilities. New J. Phys. 9:378 10.1088/1367-2630/9/10/378

[B140] Pitkow X. Sompolinsky H. Meister M. (2007). A neural computation for visual acuity in the presence of eye movements. PLoS Biol. 5:e331 10.1371/journal.pbio.0050331 18162043 PMC2222970

[B141] Poletti M. Listorti C. Rucci M. (2013). Microscopic eye movements compensate for nonhomogeneous vision within the fovea. Curr. Biol. 23, 1691–1695 10.1016/j.cub.2013.07.007 23954428 PMC3881259

[B142] Prechtl J. Cohen L. Pesaran B. Mitra P. Kleinfeld D. (1997). Visual stimuli induce waves of electrical activity in turtle cortex. Proc. Natl. Acad. Sci. U.S.A. 94, 7621–7626 10.1073/pnas.94.14.7621 9207142 PMC23872

[B143] Qu Z. Weiss J. N. Garfinkel A. (1999). Cardiac electrical restitution properties and stability of reentrant spiral waves: a simulation study. Am. J. Physiol. Heart Circul. Physiol. 276, H269–H283 9887041 10.1152/ajpheart.1999.276.1.H269

[B144] Rabinovich M. Volkovskii A. Lecanda P. Huerta R. Abarbanel H. Laurent G. (2001). Dynamical encoding by networks of competing neuron groups: winnerless competition. Phys. Rev. Lett. 87, 068102 10.1103/PhysRevLett.87.068102 11497865

[B145] Roberts J. A. Wallis G. Breakspear M. (2013). Fixational eye movements during viewing of dynamic natural scenes. Front. Psychol. 4:797 10.3389/fpsyg.2013.00797 24194727 PMC3810780

[B146] Rolfs M. (2009). Microsaccades: small steps on a long way. Vision Res. 49, 2415–2441 10.1016/j.visres.2009.08.010 19683016

[B147] Rothman J. Cathala L. Steuber V. Silver R. (2009). Synaptic depression enables neuronal gain control. Nature 457, 1015–1018 10.1038/nature07604 19145233 PMC2689940

[B148] Rucci M. Iovin R. Poletti M. Santini F. (2007). Miniature eye movements enhance fine spatial detail. Nature 447, 852–855 10.1038/nature05866 17568745

[B149] Saalmann Y. B. Kastner S. (2009). Gain control in the visual thalamus during perception and cognition. Curr. Opin. Neurobiol. 19, 408–414 10.1016/j.conb.2009.05.007 19556121 PMC3140205

[B150] Salinas E. Sejnowski T. J. (2001). Gain modulation in the central nervous system: where behavior, neurophysiology, and computation meet. Neuroscientist 7, 430–440 10.1177/107385840100700512 11597102 PMC2887717

[B151] Salinas E. Thier P. (2000). Gain modulation: a major computational principle of the central nervous system. Neuron 27, 15–21 10.1016/S0896-6273(00)00004-0 10939327

[B152] Sandstede B. Scheel A. Wulff C. (1999). Bifurcations and dynamics of spiral waves. J. Nonlin. Sci. 9, 439–478 10.1007/s003329900076

[B153] Sara S. J. Bouret S. (2012). Orienting and reorienting: the locus coeruleus mediates cognition through arousal. Neuron 76, 130–141 10.1016/j.neuron.2012.09.011 23040811

[B154] Sato T. K. Nauhaus I. Carandini M. (2012). Traveling waves in visual cortex. Neuron 75, 218–229 10.1016/j.neuron.2012.06.029 22841308

[B155] Savill N. J. Rohandi P. Hogeweg P. (1997). Self-reinforcing spatial patterns enslave evolution in a host-parasitoid system. J. theor. Biol. 188, 11–20 10.1006/jtbi.1997.0448 9299306

[B156] Schecter D. A. Nicholls M. E. Persing J. Bedard A. J. Jr. Pielke R. A. Sr. (2008). Infrasound emitted by tornado-like vortices: basic theory and a numerical comparison to the acoustic radiation of a single-cell thunderstorm. J. Atmos. Sci. 65, 685–713 10.1175/2007JAS2384.1

[B157] Schiff S. J. Jerger K. Duong D. H. Chang T. Spano M. L. Ditto W. L. (1994). Controlling chaos in the brain. Nature 370, 615–620 10.1038/370615a0 8065447

[B158] Seliger P. Tsimring L. Rabinovich M. (2003). Dynamics-based sequential memory: winnerless competition of patterns. Phys. Rev. E Stat. Nonlin. Soft Matter Phys. 67, 011905–011901 10.1103/PhysRevE.67.011905 12636530

[B159] Sendiña-Nadal I. Alonso S. Pérez-Muñuzuri V. Gómez-Gesteira M. Pérez-Villar V. Ramírez-Piscina L. (2000). Brownian motion of spiral waves driven by spatiotemporal structured noise. Phys. Rev. Lett. 84:2734 10.1103/PhysRevLett.84.2734 11017312

[B160] Serafin M. De Waele C. Khateb A. Vidal P. Mühlethaler M. (1991). Medial vestibular nucleus in the guinea-pig. Exp. Brain Res. 84, 426–433 10.1007/BF00231465 1648506

[B161] Seung H. S. (1996). How the brain keeps the eyes still. Proc. Natl. Acad. Sci. U.S.A. 93, 13339–13344 10.1073/pnas.93.23.13339 8917592 PMC24094

[B162] Seung H. S. Lee D. D. Reis B. Y. Tank D. W. (2000). Stability of the memory of eye position in a recurrent network of conductance-based model neurons. Neuron 26, 259–271 10.1016/S0896-6273(00)81155-1 10798409

[B163] Shakhnovich A. Thomas J. (1977). Micro-tremor of the eyes of comatose patients. Electroencephalogr. Clin. Neurophysiol. 42, 117–119 10.1016/0013-4694(77)90156-0 64343

[B164] Shen L. (1989). Neural integration by short term potentiation. Biol. Cybern. 61, 319–325 10.1007/BF00203180 2550085

[B165] Shlesinger M. West B. Klafter J. (1987). Lévy dynamics of enhanced diffusion: application to turbulence. Phys. Rev. Lett. 58, 1100 10.1103/PhysRevLett.58.1100 10034339

[B166] Solomon T. Weeks E. R. Swinney H. L. (1993). Observation of anomalous diffusion and lévy flights in a two-dimensional rotating flow. Phys. Rev. Lett. 71, 3975 10.1103/PhysRevLett.71.3975 10055122

[B167] Solomon T. Weeks E. R. Swinney H. L. (1994). Chaotic advection in a two-dimensional flow: Lévy flights and anomalous diffusion. Physica D 76, 70–84 10.1016/0167-2789(94)90251-8 10055122

[B168] Sparks D. L. (2002). The brainstem control of saccadic eye movements. Nat. Rev. Neurosci. 3, 952–964 10.1038/nrn986 12461552

[B169] Spauschus A. Marsden J. Halliday D. M. Rosenberg J. R. Brown P. (1999). The origin of ocular microtremor in man. Exp. Brain Res. 126, 556–562 10.1007/s002210050764 10422719

[B170] Stacey W. (2012). Better resolution and fewer wires discover epileptic spiral waves. Epilepsy Curr. 12:147 10.5698/1535-7511-12.4.147 22936887 PMC3423214

[B171] Stosiek C. Garaschuk O. Holthoff K. Konnerth A. (2003). *In vivo* two-photon calcium imaging of neuronal networks. Proc. Natl. Acad. Sci. U.S.A. 100, 7319–7324 10.1073/pnas.1232232100 12777621 PMC165873

[B172] Taniguchi D. Ishihara S. Oonuki T. Honda-Kitahara M. Kaneko K. Sawai S. (2013). Phase geometries of two-dimensional excitable waves govern self-organized morphodynamics of amoeboid cells. Proc. Natl. Acad. Sci. U.S.A. 110, 5016–5021 10.1073/pnas.1218025110 23479620 PMC3612638

[B173] Tegnér J. Compte A. Wang X.-J. (2002). The dynamical stability of reverberatory neural circuits. Biol. Cybern. 87, 471–481 10.1007/s00422-002-0363-9 12461636

[B174] Teramae J.-N. Fukai T. (2005). A cellular mechanism for graded persistent activity in a model neuron and its implications in working memory. J. Comput. Neurosci. 18, 105–121 10.1007/s10827-005-5474-6 15789172

[B175] Thiel M. Romano M. C. Kurths J. Rolfs M. Kliegl R. (2008). Generating surrogates from recurrences. Philos. Trans. R. Soc. A Math. Phys. Eng. Sci. 366, 545–557 10.1098/rsta.2007.2109 17698471

[B176] Toomre A. (1969). Group velocity of spiral waves in galactic disks. Astrophys. J. 158, 899 10.1086/150250

[B177] Tsuda I. (1991). Chaotic itinerancy as a dynamical basis of hermeneutics in brain and mind. World Futures J. Gen. Evol. 32, 167–184 10.1080/02604027.1991.9972257

[B178] Tsuda I. (2001). Toward an interpretation of dynamic neural activity in terms of chaotic dynamical systems. Behav. Brain Sci. 24, 793–809 10.1017/S0140525X01000097 12239890

[B179] Tsuda I. (2009). Hypotheses on the functional roles of chaotic transitory dynamics. Chaos 19, 015113–015113 10.1063/1.3076393 19335017

[B180] Tyukin I. Tyukina T. van Leeuwen C. (2009). Invariant template matching in systems with spatiotemporal coding: a matter of instability. Neural Netw. 22, 425–449 10.1016/j.neunet.2009.01.014 19264447

[B181] Viecelli J. (1990). Dynamics of two-dimensional turbulence. Phys. Fluids A Fluid Dyn. 2:2036 10.1063/1.857678

[B182] Viventi J. Kim D.-H. Vigeland L. Frechette E. S. Blanco J. A. Kim Y.-S. (2011). Flexible, foldable, actively multiplexed, high-density electrode array for mapping brain activity *in vivo*. Nat. Neurosci. 14, 1599–1605 10.1038/nn.2973 22081157 PMC3235709

[B183] Werner G. (2010). Fractals in the nervous system: conceptual implications for theoretical neuroscience. Front. Physiol. 1:15 10.3389/fphys.2010.00015 21423358 PMC3059969

[B184] West B. J. (2010). Fractal physiology and the fractional calculus: a perspective. Front. Physiol. 1:12 10.3389/fphys.2010.00012 21423355 PMC3059975

[B185] West B. J. Bassingthwaighte J. B. Liebovitch L. S. (1994). Fractal Physiology, Vol. 2 Oxford: Oxford University Press

[B186] Wilkinson N. Metta G. (2011). A role for cortical spiral waves in visual attention? Procedia Comput. Sci. 7, S1–S3 10.1016/j.procs.2012.01.092

[B187] Wilkinson N. Metta G. Gredeback G. (2011). Modelling the face-to-face effect: sensory population dynamics and active vision can contribute to perception of social context, in Development and Learning (ICDL), 2011 IEEE International Conference on, Vol. 2 (Frankfurt), 1–6

[B188] Williams J. Holland M. (1999). Preparing topological states of a bose–einstein condensate. Nature 401, 568–572 10.1038/44095

[B189] Winfree A. T. (1967). Biological rhythms and the behavior of populations of coupled oscillators. J. Theor. Biol. 16, 15–42 10.1016/0022-5193(67)90051-3 6035757

[B190] Winfree A. T. (1972). Spiral waves of chemical activity. Science 175, 634–636 10.1126/science.175.4022.634 17808803

[B191] Winfree A. T. (1991). Varieties of spiral wave behavior: an experimentalists approach to the theory of excitable media. Chaos 1, 303–334 10.1063/1.165844 12779974

[B192] Winfree A. T. (2001). The Geometry of Biological Time Vol. 12 New York, NY: Springer 10.1007/978-1-4757-3484-3

[B193] Wu J.-Y. Huang X. Zhang C. (2008). Propagating waves of activity in the neocortex: what they are, what they do. Neuroscientist 14, 487–502 10.1177/1073858408317066 18997124 PMC2679998

[B194] Wulff C. (1996). Theory of Meandering and Drifting Spiral Waves in Reaction-Diffusion Systems, in Doctoral thesis. Berlin: Friei Universitat Berlin

[B195] Xiao-Ping Y. Jiang-Xing C. Ye-Hua Z. Qin L. Lu-Lu W. Qian S. (2011). Spiral wave generation in a vortex electric field. Chin. Phys. Lett. 28:100505 10.1088/0256-307X/28/10/100505

[B196] Xu X. Olivas N. D. Levi R. Ikrar T. Nenadic Z. (2010). High precision and fast functional mapping of cortical circuitry through a novel combination of voltage sensitive dye imaging and laser scanning photostimulation. J. Neurophysiol. 103, 2301–2312 10.1152/jn.00992.2009 20130040 PMC2853294

[B197] Yang H. Yang J. (2007). Spiral waves in linearly coupled reaction-diffusion systems. Phys. Rev. E 76:016206 10.1103/PhysRevE.76.016206 17677542

[B198] Yu G. Ma J. Jia Y. Tang J. (2010). Dynamics of spiral wave in the coupled hodgkin–huxley neurons. Int. J. Mod. Phys. B 24, 4555–4562 10.1142/S021797921005658X

[B199] Yuan G. Xu L. Xu A. Wang G. Yang S. (2011). Spiral waves in excitable media due to noise and periodic forcing. Chaos Solit. Fract. 44, 728–738 10.1016/j.chaos.2011.06.013

[B200] Zanos S. Zanos T. P. Marmarelis V. Z. Ojemann G. A. Fetz E. E. (2012). Relationships between spike-free local field potentials and spike timing in human temporal cortex. J. Neurophysiol. 107, 1808–1821 10.1152/jn.00663.2011 22157112 PMC3331669

[B201] Zhang H. Hu B. Hu G. (2003). Suppression of spiral waves and spatiotemporal chaos by generating target waves in excitable media. Phys. Rev. E 68:026134 10.1103/PhysRevE.68.026134 14525076

[B202] Zhang H. Hu B. Hu G. Ouyang Q. Kurths J. (2002). Turbulence control by developing a spiral wave with a periodic signal injection in the complex ginzburg-landau equation. Phys. Rev. E 66:046303 10.1103/PhysRevE.66.046303 12443318

[B203] Zhang X. Li J. (2012). A novel methodology for high accuracy fixational eye movements detection, in Proc. 4th International Conference on Bioinformatics and Biomedical Technology (Singapore), 133–140

